# Pathogenomes of Atypical Non-shigatoxigenic *Escherichia coli* NSF/SF O157:H7/NM: Comprehensive Phylogenomic Analysis Using Closed Genomes

**DOI:** 10.3389/fmicb.2020.00619

**Published:** 2020-04-15

**Authors:** Emmanuel C. Nyong, Sam R. Zaia, Anna Allué-Guardia, Armando L. Rodriguez, Zaina Irion-Byrd, Sara S. K. Koenig, Peter Feng, James L. Bono, Mark Eppinger

**Affiliations:** ^1^Department of Biology, The University of Texas at San Antonio, San Antonio, TX, United States; ^2^South Texas Center for Emerging Infectious Diseases, San Antonio, TX, United States; ^3^Research Computing Support Group, The University of Texas at San Antonio, San Antonio, TX, United States; ^4^Retired, Rockville, MD, United States; ^5^United States Meat Animal Research Center, Agricultural Research Service, United States Department of Agriculture (ARS-USDA), Clay Center, NE, United States

**Keywords:** Shiga toxin (Stx) producing *Escherichia coli* (STEC), enterohemorrhagic *E. coli* (EHEC), NSF O157:H7, SF O157:NM, whole genome sequencing and typing (WGST), comparative phylogenomics, LRT (long read technology), SRT (short read technology)

## Abstract

The toxigenic conversion of *Escherichia coli* strains by Shiga toxin-converting (Stx) bacteriophages were prominent and recurring events in the stepwise evolution of enterohemorrhagic *E. coli* (EHEC) O157:H7 from an enteropathogenic (EPEC) O55:H7 ancestor. Atypical, attenuated isolates have been described for both non-sorbitol fermenting (NSF) O157:H7 and SF O157:NM serotypes, which are distinguished by the absence of Stx, the characteristic virulence hallmark of Stx-producing *E. coli* (STEC). Such atypical isolates either never acquired Stx-phages or may have secondarily lost *stx* during the course of infection, isolation, or routine subculture; the latter are commonly referred to as LST (Lost Shiga Toxin)-isolates. In this study we analyzed the genomes of 15 NSF O157:H7 and SF O157:NM strains from North America, Europe, and Asia that are characterized by the absence of *stx*, the virulence hallmark of STEC. The individual genomic basis of the Stx (−) phenotype has remained largely undetermined as the majority of STEC genomes in public genome repositories were generated using short read technology and are in draft stage, posing a major obstacle for the high-resolution whole genome sequence typing (WGST). The application of LRT (long-read technology) sequencing provided us with closed genomes, which proved critical to put the atypical non-shigatoxigenic NSF O157:H7 and SF O157:NM strains into the phylogenomic context of the stepwise evolutionary model. Availability of closed chromosomes for representative Stx (−) NSF O157:H7 and SF O157:NM strains allowed to describe the genomic basis and individual evolutionary trajectories underlying the absence of Stx at high accuracy and resolution. The ability of LRT to recover and accurately assemble plasmids revealed a strong correlation between the strains’ featured plasmid genotype and chromosomally inferred clade, which suggests the coevolution of the chromosome and accessory plasmids. The identified ancestral traits in the pSFO157 plasmid of NSF O157:H7 strain LSU-61 provided additional evidence for its intermediate status. Taken together, these observations highlight the utility of LRTs for advancing our understanding of EHEC O157:H7/NM pathogenome evolution. Insights into the genomic and phenotypic plasticity of STEC on a lineage- and genome-wide scale are foundational to improve and inform risk assessment, biosurveillance, and prevention strategies.

## Introduction

Production of a potent cytotoxin named Shiga toxin (Stx) (Trofa et al., 1999) is a virulence hallmark of STEC (O’Brien et al., 1983). Within STEC, Enterohemorrhagic *Escherichia coli* (EHEC) is a subset comprised of highly pathogenic strains, such as the O157:H7 serotype. Since its first association with human disease (Wells et al., 1983; Pennington, 2010; Sanjar et al., 2014), this once rare serotype now exists globally, and has become a public health threat for severe and widespread foodborne outbreaks. This particular lineage is the dominant causative agent of STEC outbreaks in the United States between 2007 and 2018^1^ (Mead et al., 1999; Perna et al., 2001; Rangel et al., 2005; Ferens and Hovde, 2011), and no vaccines and only a limited arsenal of therapeutic or preventive countermeasures are available (Nguyen and Sperandio, 2012). Similarly, the sorbitol fermenting (SF) O157:NM strains, that carry the H7 antigen genes, but are non-motile (NM), are a recognized pathogen that has caused many severe outbreaks in the EU (Feng et al., 2007). The ability to induce cellular damage leading to disease in humans depends on numerous virulence factors, though the most prominent is Stx, encoded on mobile temperate Stx-converting bacteriophages (Stx-phages) (Huang et al., 1987; Schmidt, 2001; Asadulghani et al., 2009; Kruger and Lucchesi, 2015). Stx_2__a_ is the most cytopathic subtype produced by this lineage, and has been associated with severe infections in humans (Tesh et al., 1993; Orth et al., 2007; Fuller et al., 2011; Russo et al., 2014). The 70-kDa toxin acts as a protein synthesis inhibitor (Melton-Celsa, 2014), causing cytopathic effects in renal endothelial cells (Konowalchuk et al., 1977; Sandvig, 2001; Tesh, 2010; Bentancor et al., 2013a, b; Lee et al., 2016). Stx is especially toxigenic to renal cells, which express the Stx-receptor globotriaosylceramide or Gb3 (Eaton et al., 2008; Shin et al., 2009; Johannes and Romer, 2010; Mauro and Koudelka, 2011; Melton-Celsa et al., 2012; Nguyen and Sperandio, 2012; Tran et al., 2014; Pacheco et al., 2018). Stx-phages are comprised of highly diverse entities (Miyamoto et al., 1999; Muniesa et al., 2004; Gamage et al., 2006; Smith et al., 2012; Kruger and Lucchesi, 2015; Shaaban et al., 2016) and their genome plasticity has been implicated to directly, or indirectly, alter Stx production (Miyamoto et al., 1999; Wagner et al., 1999; Muniesa et al., 2003; Tyler et al., 2004; Ogura et al., 2015; Yin et al., 2015). The toxigenic conversion through acquisition of Stx-phages or their secondary loss are recurring events in the STEC group (Zhou et al., 2010; Kyle et al., 2012). In the evolution of O157:H7, the acquisition of Stx-phages by *stx*-negative ancestral progenitors (Ogura et al., 2009; Zhou et al., 2010; Cooper et al., 2014; Dallman et al., 2015; Lorenz et al., 2017) occurred at various stages as described in the stepwise evolution model (Feng et al., 1998; Wick et al., 2005; Leopold et al., 2010; Rump et al., 2011; Jenke et al., 2012; Kyle et al., 2012). Carriage of Stx-phages has far-ranging evolutionary implications in the emergence, persistence, and dissemination of *stx* genes within and across STEC pathovars and prophages make up a significant portion of the EHEC O157:H7/NM chromosome resulting in widespread genetic mosaicism (Ohnishi et al., 2001; Asadulghani et al., 2009; Garcia-Aljaro et al., 2009; Kruger and Lucchesi, 2015). A single strain may carry multiple and different Stx-phages (Rusconi et al., 2016). The loss of Stx-phages can occur via induction, during infection, or even routine culturing of bacterial strains (Bielaszewska et al., 2007; Ferdous et al., 2015). Stx (−) strains have been observed in both NSF O157:H7 and SF O157:NM serogroups (Feng et al., 2001; Mellmann et al., 2005; Friedrich et al., 2007; Bielaszewska et al., 2008; Stephan et al., 2009; Themphachana et al., 2014; Uhlich et al., 2019), and other STEC (Bielaszewska et al., 2007, 2008; Tennant et al., 2009; Bentancor et al., 2010). Stx (−) strains are attenuated and also likely impacted in their overall bacterial fitness. Beyond the anthropomorphic view of Stx as key virulence factor in human STEC pathogenesis, the carriage of Stx-phages has been associated with toxin-dependent killing of predators (King et al., 1999; Eklund et al., 2002; Steinberg and Levin, 2007; Poirier et al., 2008; Lainhart et al., 2009; Los et al., 2011, 2012; Mauro and Koudelka, 2011; Stolfa and Koudelka, 2012), altered acid resistance, motility and metabolism (Robinson et al., 2006; Su et al., 2010; Veses-Garcia et al., 2015; Saile et al., 2016; Berger et al., 2019). However, we note their ability to potentially (re-)acquire Stx through Stx-phage infection (Mellmann et al., 2008). In this study, we applied LRT technology (Koren et al., 2013; Allue-Guardia et al., 2018b) and provide closed genomes and comprehensive phylogenomic analyses for representative Stx (−) NSF O157:H7 and SF O157:NM strains in our collection that tested negative for *stx*, or were cured of their Stx-phage inventory in our laboratory.

## Materials and Methods

### Strains and Characterization

A collection of 12 Stx (−) NSF O157:H7 and SF O157:NM strains of global origin was assembled, along with three laboratory-cured Stx (−) strains. Strains were isolated from different sources, including clinical cases, the animal reservoir, and produce. Except for SF O157:NM, most O157:H7 strains do not ferment sorbitol due to frameshifts in the *srlA* and *srlE* components of the glucitol/sorbitol-specific phosphotransferase system (Wick et al., 2005). Coded by the *uidA* gene, β-glucuronidase (GUD) is an inducible enzyme produced by most generic and pathogenic *E. coli*, except for O157:H7. *E. coli uidA* features both SNP and frameshift mutations but the loss of GUD production in O157:H7 is caused by a frame-shift mutations in *uidA* (+686 GG) (Feng and Lampel, 1994; Monday et al., 2001). Both of these phenotypes were key components in the development of the stepwise O157:H7 evolution model. For this reason, we specifically selected isolates that either exhibited or were negative in these metabolic phenotypes. Strain-associated metadata can be found in Supplementary Table S1. Isolates were cultured on CHROMagar-STEC and screened for the O157 antigen with the *E. coli* O157 Latex Test Kit (Thermo Fisher). The presence or absence of *stx* genes was determined by PCR (Scheutz et al., 2012) using GoTaq Green Master Mix (Promega) in a 25 μl reaction volume. O157:H7 strains that tested negative for *stx* genes were selected for complete genome sequencing.

### Genome Sequencing, Assembly, and Annotation

Strains were cultured in lysogeny broth (LB) medium (Thermo Fisher Scientific, Asheville, NC, United States) overnight at 37°C in a shaker. Total genomic DNA was extracted using the QIAamp DNA Mini Kit (Qiagen, Inc., Valencia, CA, United States) according to the manufacturer’s instructions. Nine isolates were sequenced to closure using long-read PacBio RS II technology (Supplementary Table S1). Genomic DNA was sheared into 20-kb fragments using g-TUBE (Covaris, Inc., Woburn, MA, United States). The library was prepared based on the 20-kb PacBio sample preparation protocol and sequenced using P6/C4 chemistry with either one, two or three single-molecule real-time (SMRT) cells with a 240-min collection time. The continuous long-read data were *de novo* assembled using the PacBio hierarchical genome assembly process (HGAP version 3.0) (Chin et al., 2013) with default parameters in SMRT Analysis v2.3.0, including consensus polishing with Quiver (Chin et al., 2013). In addition, some strains were sequenced using the short-read Illumina MiSeq platform (Supplementary Table S1). For Illumina sequenced isolates, a paired-end library was prepared using the NxSeq AmpFREE Low DNA Library Kit (Lucigen) with 250-bp read length and sequenced using the MiSeq Reagent kit v2 500-cycle (Illumina), following the manufacturer’s guidelines. Illumina fastq reads were trimmed and quality controlled with FastQC (Andrew, 2010) and Trim Galore (Krueger, 2017). Illumina reads were *de novo* assembled with SPAdes in the careful mode, which includes realignment (Bankevich et al., 2012). Resulting contigs were QC-filtered based on size (=<1 kb) and coverage (>=10×). Illumina reads were further used for error-correction of PacBio-only assemblies with Pilon (Walker et al., 2014). Resulting assemblies were QCed with QUAST (Gurevich et al., 2013; Mikheenko et al., 2018). The chromosomal and plasmid origins of replication^2^, *oriC* and *repA* (Gao and Zhang, 2008; Luo et al., 2018), were determined prior to annotation through the NCBI Prokaryotic Genome Annotation Pipeline (PGAP) (Tatusova et al., 2016).

### Serotype, Clade, and MLST Classification of Stx (−) NSF O157:H7 and SF O157:NM

To confirm the serotyping results of the *E. coli* O157 Latex Test Kit (Thermo Fisher), *in silico* serotyping was performed as described (Joensen et al., 2015). Clade typing was performed as originally defined by Manning et al. (2008). Clades and subgroups were assigned by *in silico* interrogation of the allelic status of 89 core genome (cg)SNPs in the assembled genomes using a custom workflow on Galaxy (Giardine et al., 2005; Goecks et al., 2010), which is informed by eight definitive polymorphic positions (Riordan et al., 2008; Yokoyama et al., 2012). The multilocus sequence types (MLST) of NSF O157:H7 and SF O157:NM cultures from our strain collection and the genomes of similar strains retrieved from GenBank were determined *in silico* with the Achtman MLST scheme, which determined the Sequence Type (ST) based on alleles in seven housekeeping genes (Wirth et al., 2006). Respective reads and/or assembled genomes were analyzed in MLST 2.0^3^ (Larsen et al., 2012) and Ridom SeqSphere+ (Junemann et al., 2013).

### Pathogenome Make-Up and Virulence Complement of Stx (−) NSF O157:H7 and SF O157:NM

The virulence and antibiotic resistance complement was identified using VirulenceFinder^4^ (Joensen et al., 2014, 2015), VDFDB (Chen et al., 2016) and ResFinder^5^ (Zankari et al., 2012; Kleinheinz et al., 2014). Prophages and plasmids were identified and distinguished from the core genomes using PHASTER (Zhou et al., 2011; Arndt et al., 2016) and PlasmidFinder^6^ (Carattoli et al., 2014). Insertion sequence (IS) elements were identified and boundaries further manually curated using ISEScan (Xie and Tang, 2017) and Iceberg (Liu et al., 2019). Genomic islands (GI) were detected with IslandViewer4 (Bertelli et al., 2017, 2018; Bertelli and Brinkman, 2018).

### Comparison of Genome Architectures and Proteome Inventory in NSF O157:H7 and SF O157:NM

To detect major structural changes in the genome architectures, sequences and gene inventories of closed chromosomes and plasmids were compared with BRIG (v0.95) (Alikhan et al., 2011) and by BLASTn/p against the non-redundant NCBI databases (Altschul et al., 1990; Camacho et al., 2009; Cock et al., 2015). BRIG visualization allowed to distinguish the core chromosome from accessory prophages and other MGEs (Alikhan et al., 2011), and further to catalog subtle polymorphisms among the carried plasmid types. To study the prevalence of the identified virulence gene complement of the core genome and carried pO157 plasmids, we used large-scale BLAST score ratio (LS-BSR) (Eppinger et al., 2010; Sahl et al., 2014; Rusconi et al., 2016) with tBLASTn (Altschul et al., 1990). For each of the proteins a BLASTp raw score was obtained for the alignment against itself (reference bit score) and the most similar protein (query bit score) in each of the genomes. The BSR value was calculated by dividing the query bit score by the reference bit score, resulting in a BSR value between 0.0 and 1.0. Proteins with a normalized BSR of <0.4 were not considered homologous. The resulting BSR matrix or alternatively the percent identities from VirulenceFinder were visualized as heatmaps with Multiple Experiment Viewer (MeV) (v.4.8) (Saeed et al., 2003).

### Shiga Toxin Status, Stx-Phage Profiling and Visualization of Phage Insertion Sites

To confirm the absence of *stx* observed from the PCR pre-screen, reads and assembled genomes were analyzed *in silico* with VirulenceFinder (Joensen et al., 2014, 2015; Kleinheinz et al., 2014) and VFDB (Chen et al., 2016). The Stx subtypes carried by the *stx*-positive reference isolates EC4115 (*stx2a*, *stx2c*) and EDL933 (*stx1*, *stx2a*) were recorded as described in Scheutz et al. (2012) and Ashton et al. (2015). STEC strains can carry Stx1 and Stx2 toxins that come in different subtypes. In NSF O157:H7 and SF O157:NM host genomes, Stx-phages target preferential loci (Kruger and Lucchesi, 2015; Yang et al., 2020). For the comparative analysis of Shiga toxin-encoding bacteriophage insertion (SBI) sites in Stx (+) and Stx (−) O157:H7, we used intact gene sequences of the NADH quinone oxidoreductase (*wrbA*), the tRNA (*argW*) (Eppinger et al., 2011b), transcriptional regulator (*yehV*) (Yokoyama et al., 2000), exonuclease (*sbcB*) (Ohnishi et al., 2002; Strauch et al., 2008), *yecE* of unknown function (Shringi et al., 2012), and oxidoreductase (Z2577) (Koch et al., 2003; Serra-Moreno et al., 2007). Unoccupied SBI were defined as those strains that showed undisrupted BLASTn alignments when queried (Altschul et al., 1990) against the O157:H7 genome assemblies. Unbiased of established phage insertion sites in O157:H7, boundaries and location of intact, partial or remnant prophages were further identified in PHASTER (Arndt et al., 2016), and genome architectures of chromosomal Stx-phage insertion sites were comprehensively analyzed and visualized using Easyfig (v2.2.2) (Sullivan et al., 2011). For each of the strains, corresponding Stx-phage insertion loci found in *stx*-positive strains EC4115 (*stx2a* at *argW* and *stx2c* at *sbcB*) and EDL933 (*stx2a* at *wrbA*, *stx1* at *yehV*) served as reference. A fragment extended by a 2 kb extension on each side was extracted and compared by BLASTn (Altschul et al., 1990).

### Comparative Phylogenomics

#### Whole Genome Alignment (WGA) Phylogeny

The Stx (−) O157:H7 genomes sequenced in this study along with Stx (−/+) NSF O157:H7 and SF O157:NM genomes downloaded from NCBI GenBank (Supplementary Table S1) and Stx (+) O157:H7 reference strain EC4115 (Eppinger et al., 2011b) were used to construct a whole genome-based phylogenetic tree. The phylogeny was inferred from whole genome alignments (WGA) using Mugsy (v1.2.3) (Angiuoli and Salzberg, 2011) and RAxML (v4.0) (Stamatakis, 2014) with 100 bootstrap replicates. The tree was visualized in Geneious 2019 (v1.2.) (Kearse et al., 2012) and decorated with strain-associated metadata in EvolView (v3) (Zhang et al., 2012; He et al., 2016; Subramanian et al., 2019).

#### Core Genome SNP Phylogeny

To compute a SNP phylogeny, we used a custom-built core genome (cg) SNP discovery pipeline described in more detail in Eppinger et al. (2010, 2011b, 2014), Rusconi et al. (2016), and Hau et al. (2018), which is implemented on the open-source web-based bioinformatics platform Galaxy (Goecks et al., 2010). The chromosomal core was defined as the set of genic and intragenic regions that are not repeated, do not contain prophages, IS elements, GIs, or other mobile genome elements (MGEs), which evolve at different rates and therefore are not indicative of evolutionary relationships. These regions were determined in the designated closed reference *E. coli* O157:H7 strain EC4115 (Eppinger et al., 2011b) as follows: Repeats with NUCmer (v3.22) (Delcher et al., 2003), prophages with PHASTER (Zhou et al., 2011; Arndt et al., 2016), and IS elements with ISFinder (Siguier et al., 2006), ISEScan (v1.7.1) (Xie and Tang, 2017), and ICEberg (Liu et al., 2019). The modular pipeline contains the following workflow steps: (i) SNP discovery and typing. When available, Illumina reads were used for read-based SNP discovery. Reads were aligned with BWA-MEM (Li and Durbin, 2009) to the designated reference genome EC4115. The resulting alignments were processed with Freebayes (v1.3.1) (Garrison and Marth, 2012) with the following threshold settings: mapping quality 30, base quality 30, coverage 10, and allelic frequency 0.75. For contig-based discovery, PacBio-only assemblies and Illumina error-corrected PacBio assemblies were aligned to the reference EC4115 chromosome (Eppinger et al., 2011b) using NUCmer, followed by SNP prediction with delta-filter and show-snps distributed with the MUMmer package (Delcher et al., 2003; Marcais et al., 2018). The resulting SNP panels for each of the query genomes were used for further processing; (ii) SNP validation and filtering. We used several SNP curation strategies detailed in our previous works (Eppinger et al., 2011b, 2014; Rusconi et al., 2016). Cataloged SNPs from each genome were merged into a single SNP panel and SNPs located within identified excluded regions were removed, as well as low quality alignments or misalignments, non-uniformly distributed regions, and InDels, as previously described (Myers et al., 2009; Morelli et al., 2010; Eppinger et al., 2014). SNPs were further curated by extracting the surrounding 40 nucleotides (nt) for each predicted SNP in the reference genome, followed by BLASTn of these fragments against the query genomes (Altschul et al., 1990). SNPs with missing information (“no hits”) or multiple hits were filtered out, as well as ambiguous nucleotides; (iii) SNP annotation and chromosomal distribution. Allelic status and chromosomal position of SNPs were recorded. To account for the biological relevance of these point mutations, polymorphisms were classified into genic or intergenic by mapping the SNPs to the reference genome annotation (Manning et al., 2008; Bono, 2009; Leopold et al., 2010; Rusconi et al., 2016). SNP-matrix tables were manipulated with Query Tabular Tool (Johnson et al., 2018). In addition, we developed a genotyper tool to provide SNP statistics reporting on the number of individual genotypes in the phylogeny; (iv) SNP phylogeny. The curated panel of high quality SNPs served as basis for phylogenetic reconstruction by maximum parsimony with PAUP (v4.0a163) (Wilgenbusch and Swofford, 2003) with 100 bootstrap replicates. The majority rule consensus SNP tree was visualized in Geneious (Kearse et al., 2012) and decorated with EvolView (Zhang et al., 2012; He et al., 2016; Subramanian et al., 2019). Calculation of the consistency index (CI) in Mesquite (Maddison and Maddison, 2016) for each SNP allowed us to identify parsimony informative SNPs and flag homoplastic SNPs, as described in our previous works (Eppinger et al., 2010, 2011b, 2014; Rusconi et al., 2016; Hau et al., 2018). This strategy was also used for the discovery of plasmid-borne SNPs referenced to closed pO157 and pSFO157 plasmids of strains EC4115 (Eppinger et al., 2011b) and 3072/96 (Brunder et al., 2006), respectively.

### Laboratory Isolation of *stx*-Cured EHEC O157:H7

Therapeutic use of certain antibiotics is known to induce Stx_2__a_ phages (Huang et al., 1987; Mead and Griffin, 1998; Zhang et al., 2000; Zimmerhackl, 2000; Wong and Brandt, 2002; McGannon et al., 2010; Wong et al., 2012; Amorim et al., 2014; Rahal et al., 2015). Strains were cured of *stx* by inducing the Stx-phages with Mitomycin C (MMC), a potent Stx_2__a_ phage inducing agent that triggers the RecA-mediated SOS-response (Fuchs et al., 1999; Kimmitt et al., 2000; Los et al., 2009; Imamovic and Muniesa, 2012). Overnight cultures of O157:H7 strains EC4115, PA2, and PA11 were diluted to an OD_600_ of 0.05 and grown to an OD_600_ of 0.3–0.5 in fresh LB medium at 37°C. MMC, a light sensitive chemical, was added at a final concentration of 0.5 μg/ml and cultures were incubated for 6 h (37°C, 200 rpm) in the dark. To recover clones that lost the *stx*, cultures were serially diluted and plated on LB agar (37°C, 16 h). Plates with 100–300 colonies were selected and the colonies were blotted to a nylon membrane (GE Healthcare Amersham Hybond-N+). Colonies that lost both the *stx1* and *2* genes were detected after colony blot hybridization (Sambrook et al., 2006) with a DIG-labeled *stx*-specific probe using the PCR DIG Synthesis and DNA Labeling and Detection Kits (Roche). The probe consisted of a 255 bp fragment amplified with primers 5′- GGCACTGTCTGAAACTGCTCC-3′ and 5′-TCGCCAGTTATCTGACATTCTG-3′ (Rajkhowa et al., 2010) following the protocol for the Phusion High-Fidelity PCR Master Mix (Thermo Fisher Scientific) in a T100 Thermal Cycler (Bio-Rad). The absence of *stx* in the recovered clones was confirmed by PCR using *stx* and insertion site-specific primers using conditions developed for the Shiga toxin-encoding bacteriophage insertion site (SBI) assay (Shaikh and Tarr, 2003; Besser et al., 2007; Scheutz et al., 2012).

## Results and Discussion

### LRT Sequencing of Stx (−) NSF O157:H7 and SF O157:NM Strains

In this study we sequenced the genomes of 14 NSF O157:H7 strains and one SF O157:H7 that are all characterized by absence of *stx*, the virulence hallmark of STEC. Isolates were analyzed along with 26 other Stx (±) NSF O157:H7 and SF O157:NM genomes available from NCBI GenBank (Supplementary Table S1). We re-sequenced the genome of SF O157:H7 strain LSU-61 to closure (Dunn et al., 2004), a presumed intermediate (Clade 9.2.36) in the stepwise evolution model (Feng et al., 1998, 2007; Wick et al., 2005; Leopold et al., 2010; Jenke et al., 2012; Kyle et al., 2012), previously only available as draft (Rump et al., 2011). Genomes of EHEC O157:H7/NM are notorious for assembling into fragmented draft genomes by the more commonly used short-read technologies (SRTs) due to the homogeneous nature of lambdoid prophages content and other repeats. Contig breaks in STEC assemblies typically occur within Stx-phages producing numerous phage-related contigs, which hinders accurate Stx-phage profiling (Asadulghani et al., 2009; Eppinger et al., 2011a, b, 2013; Smith et al., 2012; Sadiq et al., 2014; Yin et al., 2015). In response, we used LRT sequencing (English et al., 2012) on the PacBioRS II platform (Koren et al., 2013; Cooper et al., 2014; Latif et al., 2014; Loman et al., 2015; Cowley et al., 2016; Shaaban et al., 2016; Allue-Guardia et al., 2018a, b), followed by Illumina short read-error correction with Pilon (Walker et al., 2014). The resulting closed (or near-closed) genomes and plasmids (Supplementary Table S2) were subjected to high-resolution whole genome sequence typing (WGST), and provided the critical foundation to investigate the genomic basis of the strains’ atypical Stx (−) status and to determine the phylogenomic position of these atypical Stx (−) isolates within the EHEC O157:H7/NM lineage.

### Pathogenome Architecture and Virulence Profiles of Stx (−) NSF O157:H7 and SF O157:NM

**FIGURE 1:**
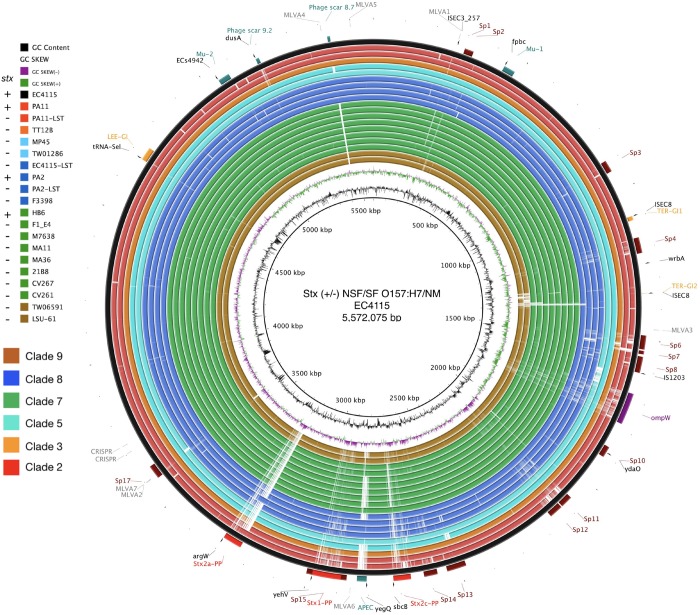
Genome architecture of Stx (±) NSF O157:H7 and SF O157:NM. Whole chromosome BRIG analysis of the genome architecture and phage inventory of the Stx (±) strains examined and referenced to the 5,572,075 bp genome of O157:H7 strain EC4115 shown in the outermost circle. The LEE-island and phage inventory are annotated, and Stx-phages are highlighted in red. Query genomes are plotted on each circle as shown in the legend. The order of genomes on each ring reflects phylogenomic position according to the stepwise model of O157:H7 evolution. Different color codes of the circles represent clade association as shown in the legend. GC-content and GC-skew of the reference are depicted in the two innermost circles, respectively.

**FIGURE 2:**
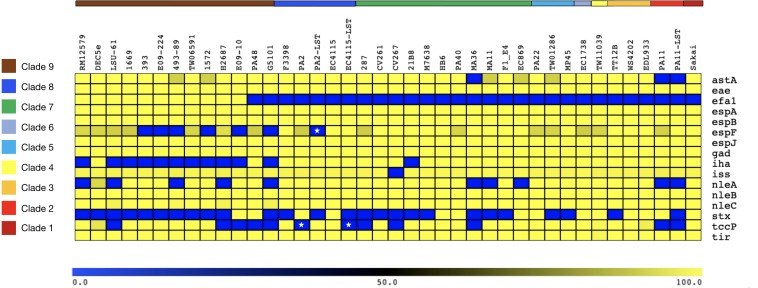
Chromosomal virulence gene inventory of Stx (±) NSF O157:H7 and SF O157:NM. Predicted virulence genes in each sampled genome were identified with VirulenceFinder (Joensen et al., 2014; Kleinheinz et al., 2014). Identities for each gene are visualized in a heatmap using MeV (Saeed et al., 2003). The order of query chromosomes reflects the phylogenomic position in the stepwise model of O157:H7 evolution. Besides the absence of Stx-phages or disruption of *stx* genes, the sampled *stx* (–) strains resemble the virulence profile of Stx (+) O157:H7/NM. Stars indicate the fragmentary status of the *tccP* and *espF* loci in the respective draft genomes.

Alterations in genome size and architecture in EHEC O157:H7/NM are driven by the individual prophage complement, particularly Stx-phages (Asadulghani et al., 2009; Eppinger et al., 2011b; Rusconi et al., 2016; Shaaban et al., 2016) and dynamics of other MGEs (Eppinger et al., 2011b; Yokoyama et al., 2011; Stanton et al., 2014; Toro et al., 2015). We compared the chromosomes of the Stx (−) NSF O157:H7 and SF O157:NM strains with BRIG (Alikhan et al., 2011) using Stx (+) strain EC4115 (*stx1*−, *stx2a*+, *stx2c*+) as reference (Eppinger et al., 2011b). As evident in Figure 1, we observed an overall genome-wide synteny of the chromosomal backbones of Stx (+) and (−) strains as has been previously established for this lineage (Eppinger et al., 2011b). The strains belong to six distinct phylogenetic clades and the comparison highlights the spectrum of genomic variations that can be found in this lineage (Manning et al., 2008). The predicted chromosomal virulence and resistance gene complement was inferred from closed genomes (Figure 2). None of the Stx (−) strains sequenced for this study encoded antibiotic resistance genes. The absence of *stx* determined by PCR-prescreen was confirmed *in silico* for all strains but HB6, which features an altered *stx2c* locus disrupted by IS insertion (Strauch et al., 2008) (Supplementary Figure S1). Apart from the absence of *stx*, these Stx (−) NSF O157:H7 and SF O157:NM strains carried the full repertoire of chromosomal and plasmid virulence determinants found in Stx (+) representatives of this lineage (Levine, 1987; Law, 2000). The *wt* progenitors and laboratory-cured LST strain pairs are indifferent in their virulence profiles. We note that *tccP*, a phage borne virulence determinant (Garmendia et al., 2004) and *espF* are present in all three *wt*/LST strain pairs, but were found fragmented on several contigs in the respective draft genome. All strains were positive for the locus of enterocyte effacement (LEE) pathogenicity Island (PI) when testing for the presence of *eae* (Yu and Kaper, 1992; Donnenberg et al., 1993; Kaper, 1998) and negative for bundle-forming pilus (*bfp*−), a fimbrial adhesin (Giron et al., 1991) common in EPEC strains. Although the *eae* gene is a virulence trait shared with EPEC, which can carry a variety of *eae* alleles, all Stx (−) NSF O157:H7 and SF O157:NM strains carried the characteristic γ-intimin allele (McGraw et al., 1999; Blanco et al., 2006) and LEE PI (Perna et al., 1998; Ferdous et al., 2015). Thus, these strains are either progenitors that gave rise to EHEC O157:H7/NM upon acquisition of Stx-phages (Kyle et al., 2012; Schutz et al., 2017), or alternatively are O157:H7/NM strains, which secondarily lost *stx* (Friedrich et al., 2007; Bielaszewska et al., 2008).

### Comparative Phylogenomics

**FIGURE 3:**
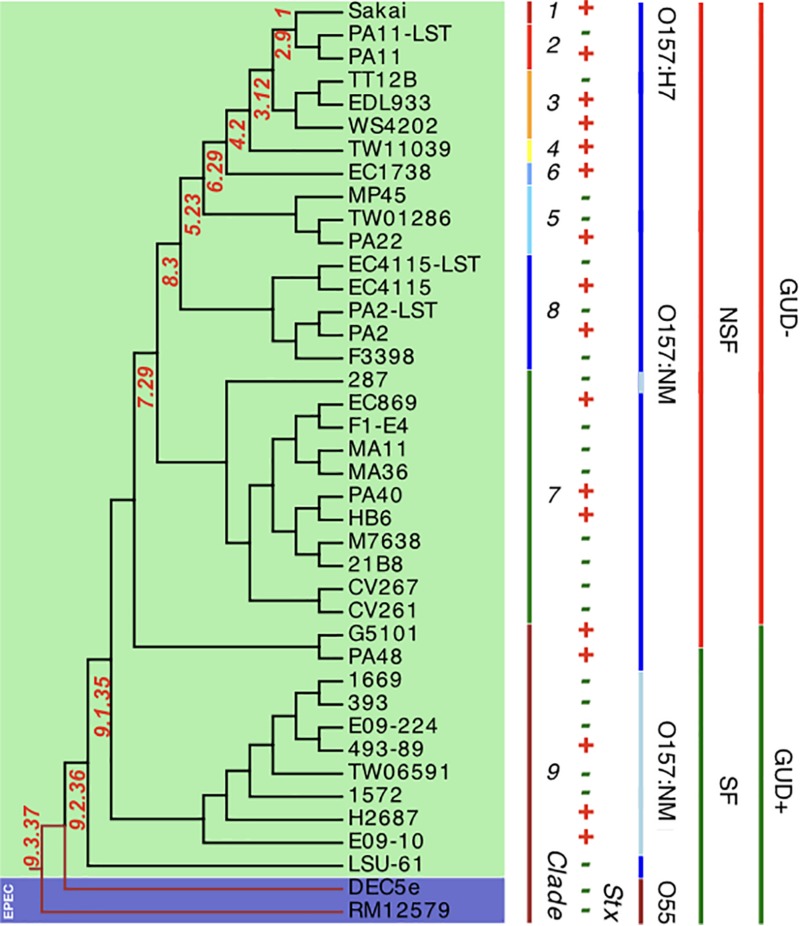
Core genome SNP phylogeny of Stx (±) O157:H7 and SF O157:NM. Comparison of 40 (LST–) EHEC O157:H7 and SF O157:NM genomes and two ancestral EPEC O55:H7 yielded a total of 7,673 SNPs (Supplementary Table S1), of which 4,109 were parsimony informative. The tree shown is the majority-consensus tree of 281 equally parsimonious trees with a CI of 0.98. Trees were recovered using a heuristic search in PAUP (Wilgenbusch and Swofford, 2003) with 100 bootstrap replicates. The tree was visualized in Geneious (Kearse et al., 2012) and decorated with strain-associated metadata in EvolView (Zhang et al., 2012; He et al., 2016), such as clade assignment, Stx-, SF-, and GUD-status. As evident from comparing the topology to the WGA tree (Supplementary Figure S2), major clusters are mirrored. The tree topology partitions the isolates into distinct phylogenetic clusters that are in accordance with the stepwise model of EHEC O157:H7/NM evolution from an EPEC O55:H7 progenitor.

*In silico* clade typing (Manning et al., 2008) assigned the Stx (−) NSF O157:H7 and SF O157:NM strains into five distinct clades (3, 5, 7, 8, and 9), indicative of evolutionary independent *stx* loss (Lacher, 2011) (Supplementary Table S1). To place the strains in the broader context of the O157:H7 stepwise evolutionary model (Feng et al., 1998; Wick et al., 2005; Leopold et al., 2010; Rump et al., 2011; Jenke et al., 2012; Kyle et al., 2012), we established a robust phylogenomic framework by constructing phylogenetic hypotheses based on WGA and *de novo* SNP discovery including representative *stx*-positive NSF O157:H7 and SF O157:NM strains (Eppinger et al., 2011b; Rusconi et al., 2016). The phylogenies show that the NM-phenotypes arose through two independent evolutionary events (Figure 3 and Supplementary Figure S2). In all analyzed SF O157:NM clade 9 isolates the NM-phenotype (Feng et al., 1996; Barkocy-Gallagher et al., 2001) is caused by a 12-bp deletion in *flhC*, the master regulator of flagellar biosynthesis (Monday et al., 2004). However, the non-motile NSF O157:NM clade 7 strain EPEC_287 (Ferdous et al., 2015) did not have this particular mutation. Instead, it features a strain-specific nsSNP in the flagellar hook length control gene *fliK* at position 616 (CCG > TCG) resulting in a (206; *P* > *S*) transition from non-polar proline to polar serine (Supplementary Table S3). Such a *P* > *S* transition, yet at another genic position, has previously been linked to altered flagellar protein secretion (Uchida et al., 2016). Further structural analysis of FliK in *E. coli* including diverse bacteria suggests functional conservation at this particular position (Marchler-Bauer et al., 2017). To determine the genetic relationships of the individual isolates at a higher level of phylogenetic accuracy and resolution, we performed a cgSNP analysis using a custom developed SNP discovery and validation pipeline described in detail in our previous works (Eppinger et al., 2010, 2011b; Rusconi et al., 2016; Hau et al., 2018). Comparison of the chromosomes yielded a total of 7,673 high-quality SNPs, of which 4,109 were parsimony informative. The resulting maximum parsimony (MP) tree using PAUP (v4a163) (Wilgenbusch and Swofford, 2003) with 100 bootstrap replicates shows bootstrap supports greater than 90 for the majority of nodes (Figure 3). As evident in both the WGA- and SNP-phylogenies, the tree topology corroborates with the phylogenetic clade placement and mirrors the general understanding of the stepwise evolutionary model of O157:H7 from an EPEC O55:H7 progenitor (Feng et al., 1998; Wick et al., 2005; Leopold et al., 2009, 2010; Rump et al., 2011; Jenke et al., 2012; Kyle et al., 2012). The phylogenomic position of LSU-61 is consistent with its proposed intermediate status between O55:H7 and the divergent NSF O157:H7 and SF O157:NM branches.

### Comprehensive Analysis of Stx-Phage Occupation Status in Stx (±) SF O157:NM and NSF O157:H7

Long-read technology provided complete prophage sequences and chromosomal context and was instrumental for accurate Stx-phage profiling (Smith et al., 2012; Yin et al., 2015; Shaaban et al., 2016; Gonzalez-Escalona et al., 2019) (Figure 1). Several Stx-phages integration sites have been established in O157:H7 (Kruger and Lucchesi, 2015). Stx_2__a_-phages preferentially target the NAD(P)H dehydrogenase *wrbA* and tRNA *argW* genes, both of which can be simultaneously occupied (Eppinger et al., 2011b; Rusconi et al., 2016). Stx_1_- and Stx_2__c_-prophages preferentially target the NADH quinone oxidoreductase *yehV* (Yokoyama et al., 2000) and transcriptional regulator *sbcB* (Strauch et al., 2008), respectively (Figure 1), though other insertion sites are known. The boundaries and location of Stx-phages were identified by PHASTER (Arndt et al., 2016), unbiased from documented EHEC phages insertions sites, and loci comprehensively analyzed and visualized in Easyfig (Sullivan et al., 2011). To represent the evolved and ancestral Stx-phage insertion states as per the stepwise model of evolution (Feng et al., 1998; Wick et al., 2005; Leopold et al., 2010; Rump et al., 2011; Jenke et al., 2012; Kyle et al., 2012), we used *stx*-positive strains EC4115 (*stx2a* at *argW*, *stx2c* at *sbcB*) (Eppinger et al., 2011b) and EDL933 (*stx1* at *yehV*) (Perna et al., 2001) and stx-negative EPEC O55:H7 strain RM12579 (Kyle et al., 2012) as references. As shown in Supplementary Figures S3–S6, a fragment corresponding to these insertion loci, extended by 2 kb on each side, was compared by BLASTn (Altschul et al., 1990). If a locus is found unoccupied in Stx (−) NSF O157:H7 or O157:NM strains, it may be indicative of secondary loss of the Stx-phage or *stx locus* (Schmidt et al., 1999; Feng et al., 2001; Koitabashi et al., 2006; Wetzel and Lejeune, 2007; Bettelheim, 2008; Rump et al., 2011; Jenke et al., 2012), or alternatively this insertion site was never targeted by Stx-phages. BLASTn comparisons of the complete Stx-phage genomes visualized in Easyfig (Sullivan et al., 2011) show largely syntenic and conserved Stx-phage architectures. As previously mentioned, strain HB6 showed a false negative *stx* PCR reaction. Genomic analysis revealed that *stx* in HB6 is disrupted by insertion sequence IS*629*, which affected the binding of the generic *stx* PCR primer, and probably renders the *stx* gene non-functional (Supplementary Figure S1) (Strauch et al., 2008). As evident in Supplementary Figures S3–S6 all Stx (−) strains examined are devoid of complete Stx-phages. The laboratory-cured LST strains showed complete loss of Stx-phages at respective Stx-phage insertion sites when compared to the *wt* strains [EC4115: *stx2a* (*argW*), *stx2c* (*sbcB*); PA2: *stx2a* (*argW*); PA11: *stx2a* (*wrbA*)]. The *sbcB* locus (Supplementary Figure S4) of strain CV261 is occupied by a phage showing homology to the 5′ and 3′ regions of the EC4115 Stx_2__c_-phage, which may suggest a deletion of the *stx*-containing region, as observed in the 2006 Spinach outbreak isolates (Eppinger et al., 2011b). By contrast, phages were detected in the majority of sampled strains occupying the *yehV* locus (Supplementary Figure S5). Comparison to the reference Stx_1_-phage of EDL933 shows partial homology but also extended dissimilar regions. It is thus unclear whether these phages are related to Stx-phages that lost *stx* or are part of the *stx*-negative phage complement of O157:H7/NM. Our profiling of phage insertion loci in closed genomes of Stx (−) strains allowed us to identify different and mechanistically unrelated scenarios, from complete absence of Stx-phages to a more confined loss or disruption of the *stx* locus by IS*629* (Eppinger et al., 2011b). This element plays a major role in shaping the STEC population structure (Ooka et al., 2009; Yokoyama et al., 2011; Stanton et al., 2014; Toro et al., 2015) including Stx-phage diversification (Eppinger et al., 2011b; Sanjar et al., 2015; Yin et al., 2015; Rusconi et al., 2016).

### Plasmid Inventory of Stx (−) SF O157:NM and NSF O157:H7 Strains

**FIGURE 4:**
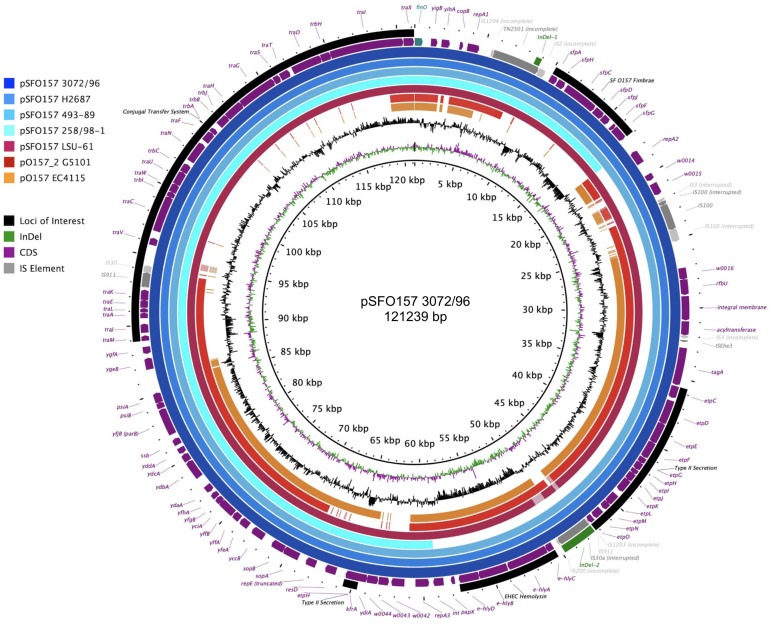
Comparison of the three major virulence plasmid variants. BRIG analysis of plasmid architecture and gene inventory of the three established virulence plasmid variants inferred from representative closed plasmids. Respective gene inventories are referenced to the largest 121-kb pSFO157 variant of strain 3072/96 (Brunder et al., 2006) shown on the outermost circle. CDS of reference plasmid are presented as purple arrows and functional annotation for loci of importance are depicted in the legend. The *finO* gene defines the origin of replication and is the designated pSFO157+1 start site. Query plasmids are plotted on each circle as shown in the legend and the order reflects phylogenomic position according to the stepwise model of O157:H7 evolution. Different color codes of the circles represent distinct plasmid types: Plasmid pSFO157 present in SF O157 strains (blue) H2687, 493-89, 258/98-1 (a 79-kb truncated variant), and O157:H7 strain LSU-61 (burgundy); and the O157:H7 plasmid types pO157 and pO157_2 from EC4115 (orange, 94,644 kb) and G5101 (red, 89,762 kb), respectively. Major differences are associated with MGEs highlighted in gray. The gene inventory of pSFO157 is distinguished from pO157 and pO157_2 by the presence of the Sfp fimbriae and an incomplete F-plasmid like conjugal transfer machinery, which accounts for the one-third larger pSFO157 plasmid size. The *hly* and *etp* operons in pSFO157-258/98-1 from the Czech Republic was lost as part of a 41,534 bp fragment via homologous recombination. GC-skew and -content of the pSFO157 3072/96 reference plasmid are depicted in the two innermost circles, respectively.

**FIGURE 5:**
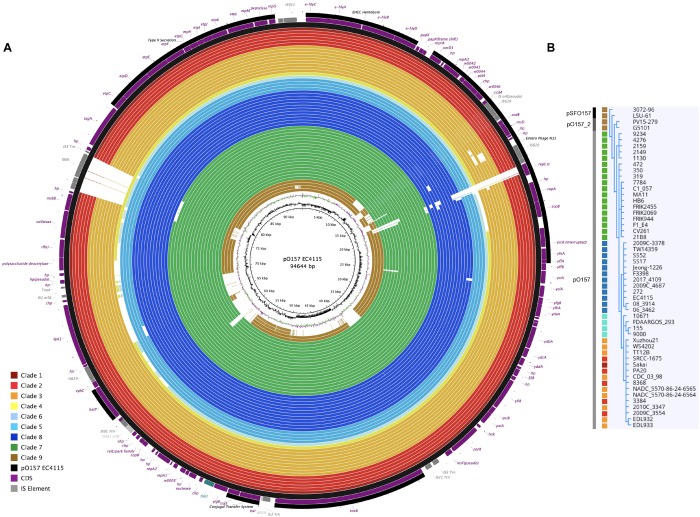
Comparison of virulence plasmid variants referenced to pO157. **(A)** BRIG visualization (Alikhan et al., 2011) of plasmid architectures and gene inventories comparing the three major virulence plasmid variants. Only draft sequences were available for clades 4 and 6 plasmids, while closed molecules represent all other plasmids. Plasmids recovered and sequenced in this study were complemented by O157:H7 and SF O157:NM plasmid sequences retrieved from GenBank, and referenced to the 94-kb pO157 plasmid type of strain EC4115, shown on the outermost circle. CDS are presented as purple arrows and functional annotation for loci of importance are depicted in the legend. The *finO* gene defines the *ori* in the reference and is designated as the +1 start site. The order of query genomes plotted in the circle reflects their respective clade association from 1 to 9 as indicated by the colored boxes in the legend. We used the pO157 plasmids of strains EDL933 and WS4202 as reference, the latter is 636 bp larger (Latif et al., 2014). Major differences are associated with MGEs, highlighted in gray. GC-content and -skew of the pO157 EC4115 reference plasmid are depicted in the two innermost circles, respectively. **(B)** SNP-derived phylogeny of three virulence plasmid types. The tree was recovered using a heuristic search in PAUP (Wilgenbusch and Swofford, 2003) with 500 bootstrap replicates, and visualized in Geneious (Kearse et al., 2012). The vertical gray bar indicates the plasmid variant and the colored boxes reflect the clade as shown in the legend.

**FIGURE 6:**
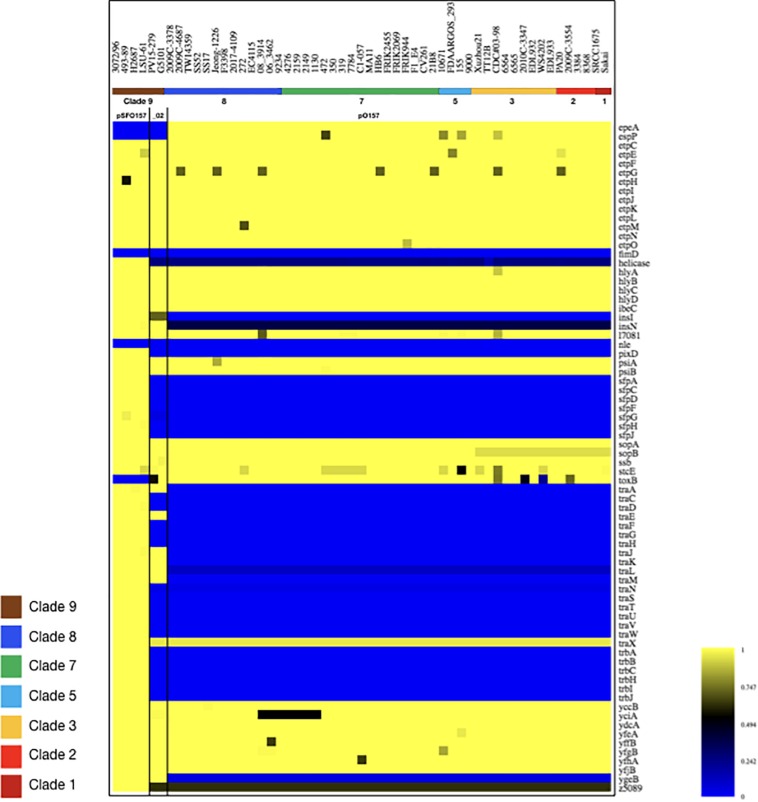
Virulence gene inventory of NSF/SF O157:H7/NM plasmids. Virulence genes were identified in pSFO157, pO157, and pO157_2 plasmid variants in representative strains 3072/96 (Brunder et al., 2006), EC4115 (Eppinger et al., 2011b), and G5101 (Rump et al., 2012) with VirulenceFinder (Joensen et al., 2014; Kleinheinz et al., 2014). Respective proteins were compared with Large-Scale Blast Score (LS-BSR) analysis (Sahl et al., 2014) and BSR values for each protein were visualized in a heatmap using MeV (Saeed et al., 2003). Query plasmids are ordered based on their phylogenomic position in the stepwise model of evolution, as shown in the legend. Values range from 0 (blue, absent) to 1 (yellow, identical). As evident in the resulting virulence profile, the gene inventory of pSFO157 is distinguished from pO157 and pO157_2 by the absence of *katP*, *etpP*, and *toxB* and conversely, by the presence of the Sfp fimbriae and an incomplete F-plasmid like conjugal transfer machinery, which account for the one third larger pSFO157 plasmid size. See also Figures 4–6 for plasticity in plasmid architectures.

Virulence plasmids play an important role in O157:H7/NM pathogenicity (Johnson and Nolan, 2009; Lim et al., 2010a, b). LRT sequencing enabled us to close plasmids for a detailed comparison of architectures and gene inventories (Gonzalez-Escalona et al., 2019). In the majority (8/12) of Stx (−) strains we detected plasmids (Supplementary Table S2). The lineage-specific pO157 plasmid (Burland et al., 1998) was present in seven strains while the larger variant pSFO157, characteristic for SF O157:NM (Brunder et al., 2006; Rump et al., 2012), was found in NSF O157:H7 strain LSU-61 (Dunn et al., 2004) (Figure 4). The latter also carries a small 37-kb plasmid with partial homology to pEC4115, described by our group as a distinguishing feature of the 2006 spinach outbreak isolates from Maine (Eppinger et al., 2011b). To support the comprehensive analysis of recovered plasmids in the context of O157:H7/NM evolution (Feng et al., 1998; Wick et al., 2005; Leopold et al., 2010; Rump et al., 2011; Jenke et al., 2012; Kyle et al., 2012), we included genomes of respective plasmid types downloaded from NCBI GenBank, focusing on closed plasmids (Hayashi et al., 2001; Perna et al., 2001; Brunder et al., 2006; Kulasekara et al., 2009; Eppinger et al., 2011b; Rump et al., 2012; Latif et al., 2014; Ferdous et al., 2015; Cowley et al., 2016; Fellner et al., 2016; Bauwens et al., 2017). Altogether, the analyzed plasmid set represents the phylogenetic diversity that can be found in O157:H7 as delineated from the clade assignment that evaluates plasticity only in chromosomal markers (Manning et al., 2008) (Supplementary Table S1). The three major plasmid types were individually analyzed by BLASTn/BLASTp (Camacho et al., 2009; Cock et al., 2015) and visualized in BRIG (Alikhan et al., 2011). The largest 121-kb pSFO157 plasmid is characteristic of SF O157:NM (Ferdous et al., 2015) strains isolated mostly in Europe (Brunder et al., 2006; Eklund et al., 2006; Friedrich et al., 2007; Buvens et al., 2009). Further, in strain G5101 (GUD+, s7/ST-65), we found the 94-kb pO157 plasmid of O157:H7 (Burland et al., 1998; Makino et al., 1998; Lim et al., 2010b; Eppinger et al., 2011b) and a distinct, slightly smaller 89,762-kb variant, termed pO157_2, which is highly related to the other two but not ancestral to pO157 (Rump et al., 2012) (Figures 4–6). We further detected a larger variant (+4,629 kb) of pO157_2 in strain PV15-279 (Ogura et al., 2018), which is larger than the one in G5101 due to the insertion of three mobile elements (IS*66*, and 2× copies of IS*629*), while G5101 codes for an additional transposase (Supplementary Figure S7C). Virulence determinants shared by these plasmids include enterohemolysin (*ehxA*) (Schmidt et al., 1995; Bielaszewska et al., 2013) and a type II secretion system (Bielaszewska et al., 2009) encoded by the *hlyCABD* and *etp* operons, respectively (Figures 4–6). Both of the latter operons were absent in pSFO157 of strain 258/98-1 from Czechia, as it was truncated (79 kb) due to a 41,534-bp deletion caused by homologous recombination, resulting in the loss of *hly* and *etp* operons (Bauwens et al., 2017). Consistent with the fact that SFO157 strains do not produce enterohemolysin. Complete pSFO157 sequences have been published from SF O157:NM strains isolated from Germany, Czechia, and Scotland, and include strains 3072/96 (Brunder et al., 2006), 258/98-1 (Bauwens et al., 2017), 493-89 (Karch et al., 1993; Rump et al., 2011), and H2687 (Rump et al., 2011), though these strains are prevalent in other European countries (Eklund et al., 2006; Friedrich et al., 2007; Buvens et al., 2009). Here we compare the architecture and content of these European-sourced pSFO157 plasmids to the sole North American representative detected in strain LSU-61. This plasmid was determined at 119,161 bp, encoding for 153 CDS. When compared to the 3072/96 pSFO157 reference plasmid (Brunder et al., 2006), we identified two InDel regions that account for the plasmid size difference of 2,078 bp, both of which are associated with IS elements (Supplementary Figure S7). Similar to the role of phages in microevolution and emergence of sublineages (Ooka et al., 2009; Eppinger et al., 2011b; Yokoyama et al., 2011; Stanton et al., 2014; Sanjar et al., 2015; Toro et al., 2015; Yin et al., 2015; Rusconi et al., 2016), mobile elements are also major drivers of plasmid diversification and are responsible for the observed differences in pSFO157 plasmid size. InDel-1 is located within the boundaries of transposable element Tn*2501* (Michiels et al., 1987) present in the LSU-61 and 3072/96 plasmids, though the LSU-61 variant is 400 bp larger (Supplementary Figure S7A). The likely intact 3,815 bp transposon of LSU-61 shares a 100% coverage and 99.5% sequence similarity to respective loci on bovine *E. coli* plasmids of strains GB089-pCFSAN004181P (CP012499) and a serogroup O168 strain 09-00049-pCFSAN004180G (CP012500), while in 3072/96 this InDel results in an N-terminal truncated transposase and recombinase. Altogether this suggests an ancestral status of LSU-61 and secondary decay in the pSFO157 plasmids from 3072/96, 493/89, and H2687, provided additional evidence for its intermediate status (Feng et al., 1998). To the contrary, LSU-61 lacks a nested mobile element of 2,080 bp comprised of IS*30*/I*S911* (Supplementary Figure S7B). Plasticity in the transfer machinery accounts for the observed variation in plasmid size of pSFO157, pO157, and pO157_2 (Fernandez-Lopez et al., 2016). The pSFO157 plasmid found in SF O157:NM strains resembles most closely the conjugal transfer region of the F-plasmid (Frost et al., 1994), with deletions in the conjugal transfer machinery (*traP*, *trbD trbG*, and *traV*) mediated by IS insertions. All plasmids share the F-plasmid leading region (*yccB-parB*). However, compared to the ancestral pSFO157 state, both pO157 and pO157_2 secondarily lost parts of the extensive transfer machinery and flanking regions, yet to a different extent: pO157 lacks the entire transfer region between *parB* and *traX*, while pO157_2 retains transfer genes (*traM-K*). The corresponding region in both pO157 and pO157_2 carries *toxB* (Tatsuno et al., 2001) nested between IS elements (IS*3*, IS*21*, and IS*629*) (Figure 6). Both pO157/pO157_2 also feature a truncated *traI* locus neighboring *traX*. Plasmid pSFO157 is further distinguished from pO157 and pO157_2 by the Sfp fimbriae encoded by the *sfpAHCDJFG* operon (Brunder et al., 2001), which functions as adhesin in SFO157 (Musken et al., 2008) (Figure 6) and is also present in O165:H25/NM strains (Bielaszewska et al., 2009). In contrast, pSFO157 lacks the catalase peroxidase KatP (Brunder et al., 1996) and the serine protease EspP (Brunder et al., 1997), present in both pO157_2 and pO157. In the truncated pSFO157 variant 258/98-1 the deletion of a 41,534 bp region via homologous recombination resulted in the loss of *hly* and *etp* operons (Bauwens et al., 2017). Referenced based SNP discovery using SF O157:NM strain 3072/96 (Brunder et al., 2006) identified a total of 38 SNPs within pSFO157-type plasmids. LSU-61 features 34 strain-specific SNPs on its plasmid, which corroborates with its distinct phylogenetic position (Figure 3 and Supplementary Figure S2). The majority of SNPs previously reported by Rump et al. (2012) were confirmed, though three were rejected during QC (Supplementary Table S6).

### Correlation of Detected Plasmid Genotypes and Stepwise Evolutionary Model

The application of LRT sequencing allowed us to accurately assemble six pO157- and one pSFO157-type plasmid to closure that complement the relatively few closed O157:H7/NM virulence plasmids deposited in NCBI GenBank. This sequence base opened an avenue to investigate whether pSFO157 and pO157 plasmids and the chromosomes evolve in parallel and how the individual plasmid genotypes relate to the stepwise model of evolution (Feng et al., 1998; Wick et al., 2005; Leopold et al., 2010; Rump et al., 2011; Jenke et al., 2012; Kyle et al., 2012). This plasmid set now encompasses a wide phylogenetic space as delineated from chromosomal based clade assignment (Manning et al., 2008) (Supplementary Table S1). In Figure 5, we plotted the closed plasmid types according to their phylogenetic position in the stepwise emergence of O157:H7 (Supplementary Table S1). As evident in Figure 5, we observed a stable evolutionary relationship between the host chromosome delineated clade and respective carried plasmid genotype. We note here that although these plasmids carry different portions of the conjugal transfer machinery, neither type is considered to be transferable on its own due to the absence of a functional *traB* gene. This locus is critical for F pilus assembly and is either truncated (41%) in pSFO157 and pO157_2 or absent in pO157 (Kim et al., 1993; Fernandez-Lopez et al., 2016). Comprehensive analysis of these on average 94-kb plasmids (Figure 5) showed that InDels are mostly associated with mobile insertion elements, but also other loci dispersed throughout the plasmid (e.g., pseudogene *insF-hp*), some of which are clade-defining signatures in plasmids (e.g., clade 3). IS elements have been utilized for chromosomal and also Stx-phage subtyping in O157:H7 (Eppinger et al., 2011b; Yokoyama et al., 2011; Stanton et al., 2014; Toro et al., 2015). The mosaic-like composition of the large pO157 virulence plasmid types, as reflected by carriage of a number of replication-associated genes and mobile genetic elements driving plasmid diversification, clearly suggests complex evolutionary origins resulting in distinct coding capacity and architectures. In this study, we identified ancestral and derived plasmid characteristics that opens the avenue for refined assay development in support of established chromosomal typing schemas (Rusconi and Eppinger, 2016). The pSFO157, pO157, and pO157_2 plasmids are related, though the latter was found to be non-ancestral to pO157 (Rump et al., 2012) (Figures 4, 5). To further elucidate the genetic relationships of the three distinct pSFO157, pO157, and pO157_2 variants, we performed SNP discovery in 54 representative plasmids. The tree was inferred from 87 SNPs, of which 37 are parsimony informative (Supplementary Table S7). The resulting topology places the plasmid variants at distinct phylogenetic positions, and further clearly shows that the overall plasmids cluster in accordance to their chromosomally inferred clade type (Figure 5B). Some of these SNP signatures were found to be clade specific (Supplementary Table S7). Altogether, our genotypic data from this limited sample suggests a stable relationship between the bacterial chromosome and carried virulence plasmids in NSF O157:H7 and SF O157:NM strains. We also investigated the prevalence, gene inventory and structural organization of pEC4115 by BRIG comparison (Alikhan et al., 2011) to related *Escherichia* and *Salmonella* plasmids, as determined by BLASTn-inferred nucleotide sequence similarities (Altschul et al., 1990). We detected this plasmid in LSU-61 and strains (FDAARGOS293, FRIK2455, SS17, 144 and 155) (Supplementary Figure S8). This relatively small 37,452 bp plasmid was sequenced previously by our group and identified as a distinguishing feature of the 2006 O157:H7 spinach outbreak isolates from Maine (Eppinger et al., 2011b). Noteworthy, it carries a number of conjugal transfer genes; however, these genes are unrelated to the *tra* machinery on pSFO157, and to remnant systems in pO157 and pO157_2.

### Comparison of Accuracy From NGS Short- and Long-Read Technology

**FIGURE 7:**
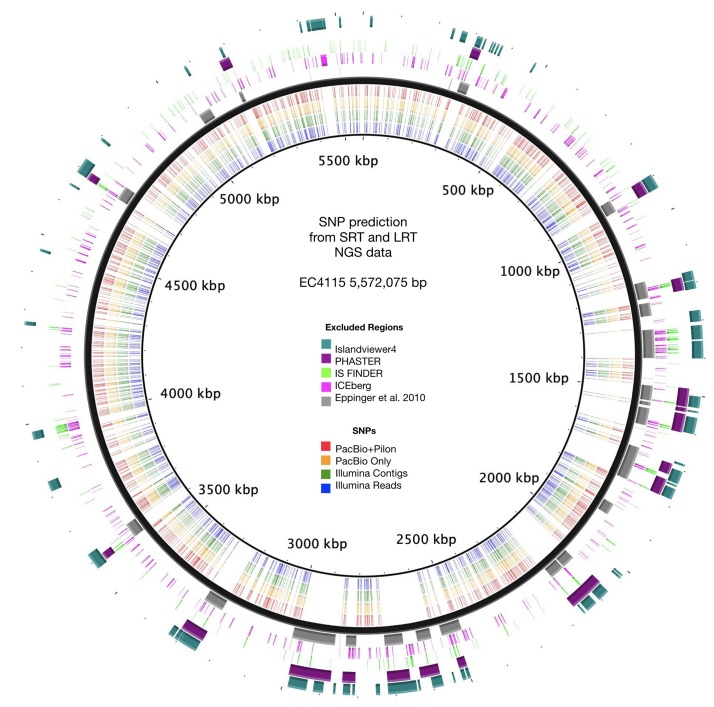
SNP prediction accuracy from short and long-read NGS sequence data. Predicted SNPs for each of the four NGS sequence data sets are plotted according to the position in the reference chromosome EC4115. SNP deserts correlated with the identified and excluded mobilome and repeat regions.

**FIGURE 8:**
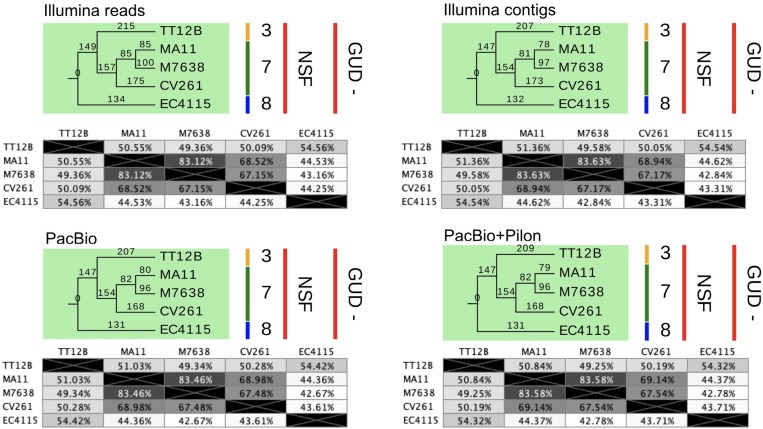
SNP statistics from NGS short- and long-read sequence data. The trees shown are the majority-consensus trees of equally parsimonious trees inferred from all four NGS sequence data sets. Trees were recovered using a heuristic search in PAUP (Wilgenbusch and Swofford, 2003) with 500 bootstrap replicates and each of the computed trees features an overall CI value > = 0.997. The numbers in the pair-wise distance matrices represent percent identity of bases, which are identical. The trees were visualized in Geneious (Kearse et al., 2012) and decorated with strain-associated metadata in EvolView (Zhang et al., 2012; He et al., 2016), such as clade assignment and metabolic properties. As evident when comparing the tree topologies and statistics, all NGS datasets provide a robust basis for phylogenetic studies and carry the identical phylogenetic information.

As evident from our analysis of the Stx-occupation status, closed genomes are key for detailed comparison of structural and genetic polymorphisms (Shaaban et al., 2016). We further used the Illumina and PacBio data set to investigate the SNP accuracy as inferred from NGS short- and long-read data. Using read- and contig-based workflows in Galaxy for reference-based SNP discovery against EC4115 (clade 8.32) (Rusconi et al., 2016), we comprehensively analyzed SNP prediction accuracy in clade 7.29 strains CV261, MA11, MA7638, and clade 3.12 strain TT12B (Feng et al., 2001). The SNP discovery was performed on four NGS data sets: Illumina reads, Illumina assemblies, PacBio assemblies, and in addition Illumina error-corrected PacBio assemblies in Pilon (Supplementary Table S1) (Walker et al., 2014). The total number of predicted unique SNPs inferred from NGS short- and long-read sequence data is 1117 with a range between 1064 (PacBio assembly) and 1096 (Illumina reads) with a standard deviation of 12.97 (Supplementary Table S4 and Figure 7). The effect on coding capacity of predicted SNPs is summarized in Supplementary Table S5. The Pilon error-corrected PacBio assemblies show only minimal changes when compared to the PacBio only assemblies with a deviation in predicted SNP numbers of 1.41. As evidenced in Figure 8, all four analyzed NGS datasets provide a robust basis for phylogenetic studies and all trees transport the identical phylogenetic information. The CI index can be an indication of a highly accurate tree and was examined for each individual SNP in the four NGS datasets (Eppinger et al., 2014). The CI for SNP prediction from Illumina and error-corrected PacBio assemblies is 1. As shown in Supplementary Table S4, a single SNP in the PacBio only assembly has a CI < 1, while three SNPs with CI < 1 were recorded from discovery in Illumina reads. Among the latter, three SNPs were called in Illumina reads only, thus the remaining 40 SNPs only predicted from Illumina reads are considered high-quality SNPs with a CI of 1. Overall, our data suggest that while Illumina read data can provide additional and phylogenetically relevant SNPs, error correction of long-reads by Illumina reads is optional.

## Conclusion

Taken our findings together, this study highlights the utility of LRT for advancing our understanding of the EHEC O157:H7/NM pathogenome evolution. The generated long-read data was critical to put these atypical Stx (−) strain into the phylogenomic context of the stepwise evolutionary model for O157:H7/NM (Feng et al., 1998; Wick et al., 2005; Leopold et al., 2009, 2010; Jenke et al., 2012; Kyle et al., 2012). Availability of closed genomes recovered through LRT allowed us to describe the underlying genomic basis and evolutionary scenarios for the absence, acquisition and loss of *stx*. Analysis of the closed virulence plasmids showed a strong correlation between plasmid genotypes and chromosomally inferred clades (Manning et al., 2008), which may indicate coevolution of the chromosome and carried plasmids. Accessory plasmids may get lost during laboratory cultivation or are often only recovered in fragments by SRT. Typing efforts are thus often focused on stable chromosomal markers (Yang et al., 2004; Manning et al., 2008); however, our data suggest that plasmid information is valuable to complement chromosomal markers and further refine the stepwise model. Insight into the strain-to-strain plasticity in the pathogenome and pathotypes of Stx (−) and (+) NSF/SF O157:H7/NM strains is foundational to improve risk assessment, biosurveillance and prevention strategies (Eppinger and Cebula, 2015; Werber and Scheutz, 2019).

## Data Availability Statement

The sequence data sets generated and analyzed in this study have been deposited in the Short Read Archive (SRA) and the Whole Genome Shotgun Repository at NCBI. Accessions for reads, annotated genomes and plasmids together with strain-associated metadata are provided in Supplementary Tables S1, S2.

## Ethics Statement

An ethical review process for the bacterial strains sequenced and analyzed in this study was not required. The animal strains 21B8, F1-E4, HB6, MP45, CV261, CV267, and M7638, sequenced in this study, were all taken at facilities that are privately owned and permission to collect freshly deposited bovine-, respectively, swine feces, from the pen surfaces was granted by the owners. Isolation history and strain-associated metadata for strains that were (re-) sequenced for this study, such as TT12B and LSU-61, as well as the wt strains from which the Stx-cured strains EC4115-LST, PA2-LST, and PA11-LST are derived, have been previously published.

## Author Contributions

ME conceived and designed the experiments. EN, SZ, AA-G, ZI-B, SK, PF, JB, and ME analyzed the data. PF contributed strain material. JB provided PacBio sequence data. AR provided computational support. ME and SK wrote the manuscript.

## Conflict of Interest

The authors declare that the research was conducted in the absence of any commercial or financial relationships that could be construed as a potential conflict of interest. The handling editor declared a past co-authorship with one of the authors ME.

## Footnotes

1 https://www.cdc.gov/ecoli/outbreaks.html

2 http://origin.tubic.org/Ori-Finder/

3 https://cge.cbs.dtu.dk/services/MLST/

4 https://cge.cbs.dtu.dk/services/VirulenceFinder/

5 https://cge.cbs.dtu.dk/services/ResFinder/

6 https://cge.cbs.dtu.dk/services/PlasmidFinder/

## Supplementary Material

FIGURE S1 Comparison of Stx_2__c_-prophages in strains EC4115 and HB6. The boundaries of the Stx_2__c_-phage inserted at the *sbcB* locus were identified in strain HB6 using PHASTER (Arndt et al., 2016). Phage architecture and gene inventories were compared to the corresponding locus in Stx_2__c_-positive strain EC4115 (Eppinger et al., 2011b) by BLASTn and visualized in Easyfig (Sullivan et al., 2011). Comparison revealed disruption of the *stx* locus in HB6 by IS629 insertion. Arrows represent predicted CDSs and gray shaded regions connect homologous sequences. Core chromosome genes flanking the phage are colored in teal. Functional annotations of predicted phage genes are shown in the legend.

FIGURE S2 Whole genome phylogeny of Stx (±) NSF O157:H7 and SF O157:NM. Genomes of 40 Stx (−) NSF O157:H7 and SF O157:NM and two progenitor EPEC O55:H7 strains, 15 of which were sequenced for this study, were aligned with Mugsy (Angiuoli and Salzberg, 2011). The phylogenetic tree with representative strains for all nine established phylogenetic clades was inferred using RAxML with a 100 bootstrap replicates (Rump et al., 2011). The majority consensus tree was visualized in Geneious (Kearse et al., 2012) and decorated with strain-associated metadata in Evolview (Zhang et al., 2012; He et al., 2016). The tree topology partitions the isolates into distinct phylogenetic clusters that corroborate with the stepwise model of O157:H7 evolution from an EPEC O55:H7 progenitor. Stx (−) strains examined clustered with NSF O157:H7 and SF O157:NM, indicative of their evolutionary independent Stx-phage or *stx* loss.

FIGURE S3 Comparison of Stx-phage occupation status at the *wrbA* locus. Easyfig comparison (Sullivan et al., 2011) of *wrbA*, a preferred target locus for Stx_2__a_-phage insertion. A fragment extended by 2 kb on each side of corresponding *wrbA* loci was extracted and compared by BLASTn (Altschul et al., 1990). Comparison of the reference Stx_2__a_-phage locus in strain EDL933 (Perna et al., 2001) and sampled Stx (−) O157:H7 strains reveals unoccupied *wrbA* loci. Arrows represent predicted CDSs and gray shaded regions connect homologous sequences. Core chromosome genes flanking the phage are colored in teal. Functional annotations of predicted phage genes are shown in the legend.

FIGURE S4 Comparison of Stx-phage occupation status at the *sbcB* locus. Easyfig comparison (Sullivan et al., 2011) of *sbcB*, a preferred target locus for Stx_2__c_-phage insertion. A fragment extended by 2 kb on each side of the corresponding *sbcB* loci was extracted and compared by BLASTn (Altschul et al., 1990). Comparison between the Stx_2__c_-phage locus in EC4115 (Eppinger et al., 2011b) and sampled Stx (−) strains reveals unoccupied *sbcB* loci with the notable exception of strain CV261 that carried phage remnants. Arrows represent predicted CDSs and gray shaded regions connect homologous sequences. Core chromosome genes flanking the phage are colored in teal. Functional annotations of predicted phage genes are shown in the legend.

FIGURE S5 Comparison of Stx-prophage occupation status at the *yehV* locus. Easyfig comparison (Sullivan et al., 2011) of *yehV*, a preferred target locus for Stx_1_-phage insertion. A fragment extended by 2 kb on each side of the corresponding *yehV* loci was extracted and compared by BLASTn (Altschul et al., 1990). Comparison between the reference Stx1-phage locus in EDL933 (Perna et al., 2001) and sampled Stx (−) strains reveals prophage sequences at *yehV* for the majority of strains, while this locus is unoccupied in TT12B and LSU-61. Arrows represent predicted CDSs and gray shaded regions connect homologous sequences. Core chromosome genes flanking the phage are colored in teal. Functional annotations of predicted phage genes are shown in the legend.

FIGURE S6 Comparison of Stx-prophage occupation status at the *argW* locus. Easyfig comparison (Sullivan et al., 2011) of *argW*, a preferred target locus for Stx_2__a_-phage insertion. A fragment extended by 2 kb on each side of the corresponding *argW* loci was extracted and compared by BLASTn (Altschul et al., 1990). Comparison between the reference Stx_2__a_-phage locus in EC4115 (Eppinger et al., 2011b) and sampled Stx (−) strains reveals unoccupied *argW* loci. Arrows represent predicted CDSs and gray shaded regions connect homologous sequences. Core chromosome genes flanking the phage are colored in teal. Functional annotations of predicted phage genes are shown in the legend.

FIGURE S7 Length polymorphisms in plasmid pSFO157 of LSU-61 and 3072/96. Plasmid architecture and gene inventories were compared with Mauve (Darling et al., 2010), and respective annotations mapped in Geneious (Maddison and Maddison, 2016). IS elements are a major driver and hotspots for pSFO157 and pO157_2 plasmid diversification. The pSFO157 plasmid of strains LSU-61 and 3072/96 differs by 2,078 bp, **(A)** InDel-1 is located within the boundaries of a Tn*2501* element resembling the 400 bp larger ancestral variant in LSU-61. **(B)** To the contrary, LSU-61 lacks a nested composite IS30/IS911 element of 2,080 bp. **(C)** InDels in pO157_2 of strains G5101and PV15-279, all of which are associated with insertion sequences.

FIGURE S8 Comparison of pEC4115 with phylogenetically related plasmids. BRIG analysis of the plasmid architecture and gene inventory of pEC4115 and related *Escherichia* and *Salmonella* plasmids, as determined by BLASTn inferred nucleotide sequence similarities (Altschul et al., 1990). Respective gene inventories are referenced to the 37,452 bp pEC4115 plasmid in the outermost circle. CDS are shown as purple arrows and the functional annotation of loci of importance are depicted in the legend. The order of plasmids on each ring reflects the sequence similarity of query plasmids as reported by BLASTn and circle colors identify plasmid source as shown in the legend. Depicted plasmids show a considerable range in plasmid size from 34,276 bp (*E. coli* O103 FHI21) to 65,419 bp (*Salmonella enterica*). Only plasmids from SS17, and human fecal isolates O181:H49 2014C-3097 and O103 FHI21 show homology to the pEC4115 origin of replication. Noteworthy, the pEC4115 plasmid also codes conjugal transfer genes. GC-skew and GC-content of the pEC4115 reference plasmid are depicted in the two innermost circles, respectively.

TABLE S1 Strain associated metadata of isolates investigated in this study.

TABLE S2 Plasmids and associated metadata of isolates investigated in this study.

TABLE S3 Core genome SNPs in investigated Stx (±) NSF O157:H7 and SF O157:NM.

TABLE S4 SNP predictions from NGS short and long-read sequence data.

TABLE S5 SNP statistics from NGS short and long-read sequence data.

TABLE S6 SNPs in pSFO157-variant plasmids referenced to strain 3072/96.

TABLE S7 SNPs in pO157-variant plasmids referenced to strain EC4115.
