# Supplementary figures and images for: Pathogenomes of Atypical Non-shigatoxigenic Escherichia coli NSF/SF O157:H7/NM: Comprehensive Phylogenomic Analysis Using Closed Genomes
Source: Front Microbiol. 2020 Apr 15;11:619. doi: 10.3389/fmicb.2020.00619 (PMC7175801; doi:10.3389/fmicb.2020.00619)

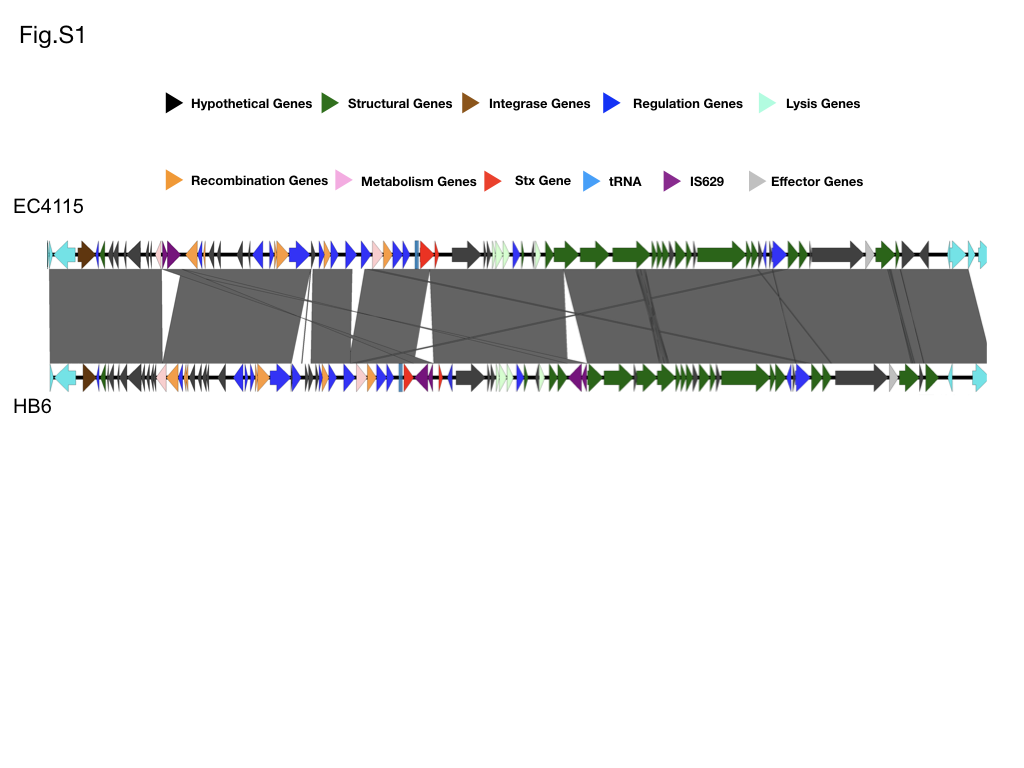

Supplement: FIGURE S1 — Comparison of Stx2c-prophages in strains EC4115 and HB6. The boundaries of the Stx2c-phage inserted at the sbcB locus were identified in strain HB6 using PHASTER (Arndt et al., 2016). Phage architecture and gene inventories were compared to the corresponding locus in Stx2c-positive strain EC4115 (Eppinger et al., 2011b) by BLASTn and visualized in Easyfig (Sullivan et al., 2011). Comparison revealed disruption of the stx locus in HB6 by IS629 insertion. Arrows represent predicted CDSs and gray shaded regions connect homologous sequences. Core chromosome genes flanking the phage are colored in teal. Functional annotations of predicted phage genes are shown in the legend. [file Image_1.TIFF]

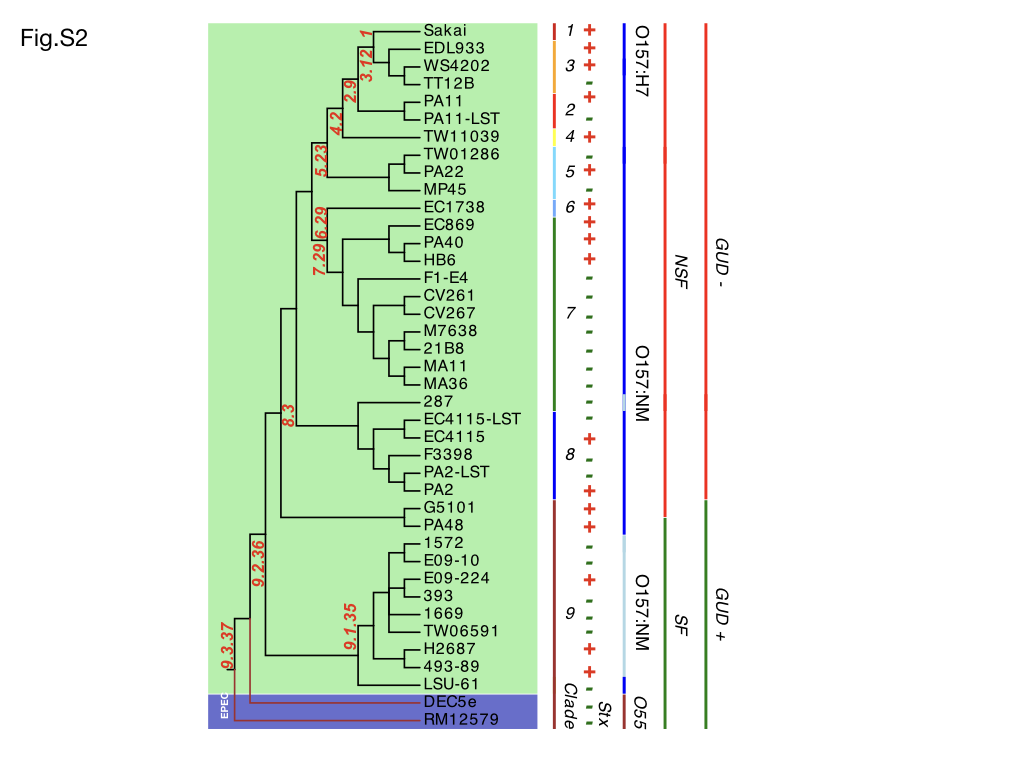

Supplement: FIGURE S2 — Whole genome phylogeny of Stx (±) NSF O157:H7 and SF O157:NM. Genomes of 40 Stx (−) NSF O157:H7 and SF O157:NM and two progenitor EPEC O55:H7 strains, 15 of which were sequenced for this study, were aligned with Mugsy (Angiuoli and Salzberg, 2011). The phylogenetic tree with representative strains for all nine established phylogenetic clades was inferred using RAxML with a 100 bootstrap replicates (Rump et al., 2011). The majority consensus tree was visualized in Geneious (Kearse et al., 2012) and decorated with strain-associated metadata in Evolview (Zhang et al., 2012; He et al., 2016). The tree topology partitions the isolates into distinct phylogenetic clusters that corroborate with the stepwise model of O157:H7 evolution from an EPEC O55:H7 progenitor. Stx (−) strains examined clustered with NSF O157:H7 and SF O157:NM, indicative of their evolutionary independent Stx-phage or stx loss. [file Image_2.TIFF]

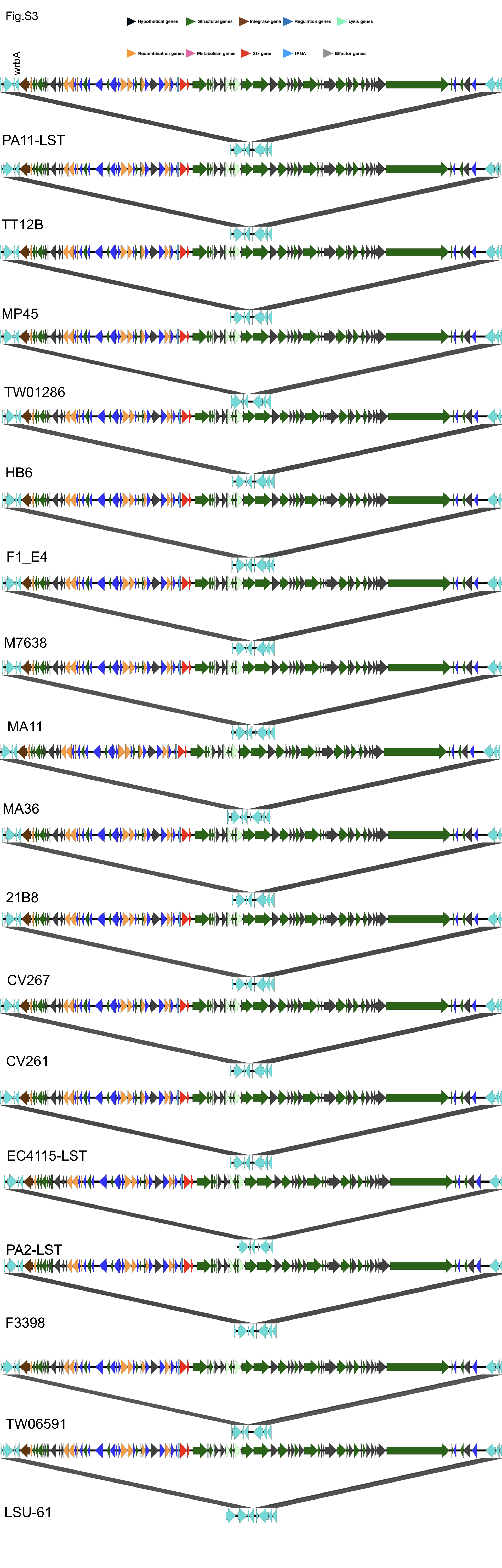

Supplement: FIGURE S3 — Comparison of Stx-phage occupation status at the wrbA locus. Easyfig comparison (Sullivan et al., 2011) of wrbA, a preferred target locus for Stx2a-phage insertion. A fragment extended by 2 kb on each side of corresponding wrbA loci was extracted and compared by BLASTn (Altschul et al., 1990). Comparison of the reference Stx2a-phage locus in strain EDL933 (Perna et al., 2001) and sampled Stx (−) O157:H7 strains reveals unoccupied wrbA loci. Arrows represent predicted CDSs and gray shaded regions connect homologous sequences. Core chromosome genes flanking the phage are colored in teal. Functional annotations of predicted phage genes are shown in the legend. [file Image_3.JPEG]

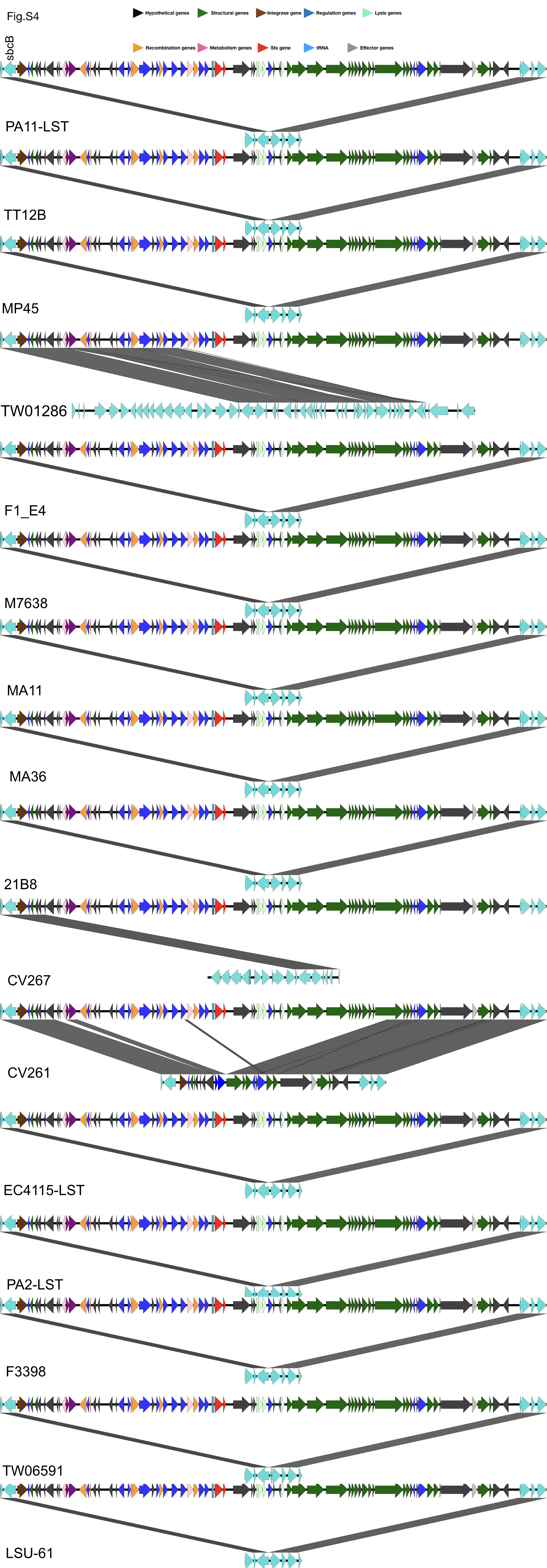

Supplement: FIGURE S4 — Comparison of Stx-phage occupation status at the sbcB locus. Easyfig comparison (Sullivan et al., 2011) of sbcB, a preferred target locus for Stx2c-phage insertion. A fragment extended by 2 kb on each side of the corresponding sbcB loci was extracted and compared by BLASTn (Altschul et al., 1990). Comparison between the Stx2c-phage locus in EC4115 (Eppinger et al., 2011b) and sampled Stx (−) strains reveals unoccupied sbcB loci with the notable exception of strain CV261 that carried phage remnants. Arrows represent predicted CDSs and gray shaded regions connect homologous sequences. Core chromosome genes flanking the phage are colored in teal. Functional annotations of predicted phage genes are shown in the legend. [file Image_4.JPEG]

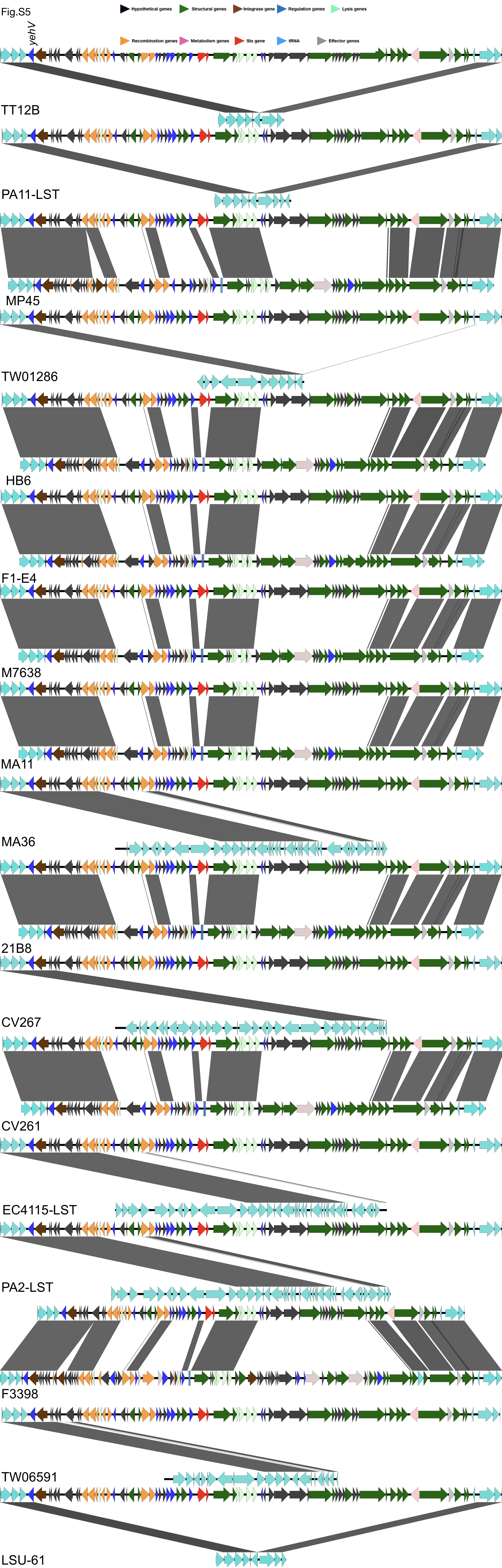

Supplement: FIGURE S5 — Comparison of Stx-prophage occupation status at the yehV locus. Easyfig comparison (Sullivan et al., 2011) of yehV, a preferred target locus for Stx1-phage insertion. A fragment extended by 2 kb on each side of the corresponding yehV loci was extracted and compared by BLASTn (Altschul et al., 1990). Comparison between the reference Stx1-phage locus in EDL933 (Perna et al., 2001) and sampled Stx (−) strains reveals prophage sequences at yehV for the majority of strains, while this locus is unoccupied in TT12B and LSU-61. Arrows represent predicted CDSs and gray shaded regions connect homologous sequences. Core chromosome genes flanking the phage are colored in teal. Functional annotations of predicted phage genes are shown in the legend. [file Image_5.JPEG]

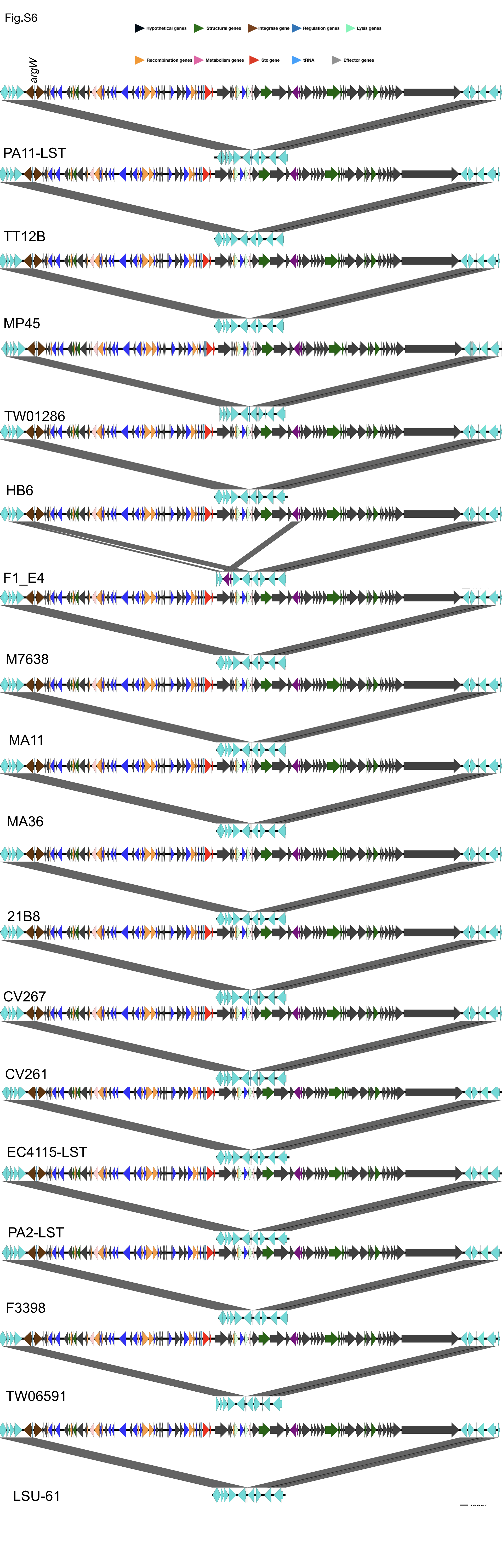

Supplement: FIGURE S6 — Comparison of Stx-prophage occupation status at the argW locus. Easyfig comparison (Sullivan et al., 2011) of argW, a preferred target locus for Stx2a-phage insertion. A fragment extended by 2 kb on each side of the corresponding argW loci was extracted and compared by BLASTn (Altschul et al., 1990). Comparison between the reference Stx2a-phage locus in EC4115 (Eppinger et al., 2011b) and sampled Stx (−) strains reveals unoccupied argW loci. Arrows represent predicted CDSs and gray shaded regions connect homologous sequences. Core chromosome genes flanking the phage are colored in teal. Functional annotations of predicted phage genes are shown in the legend. [file Image_6.JPEG]

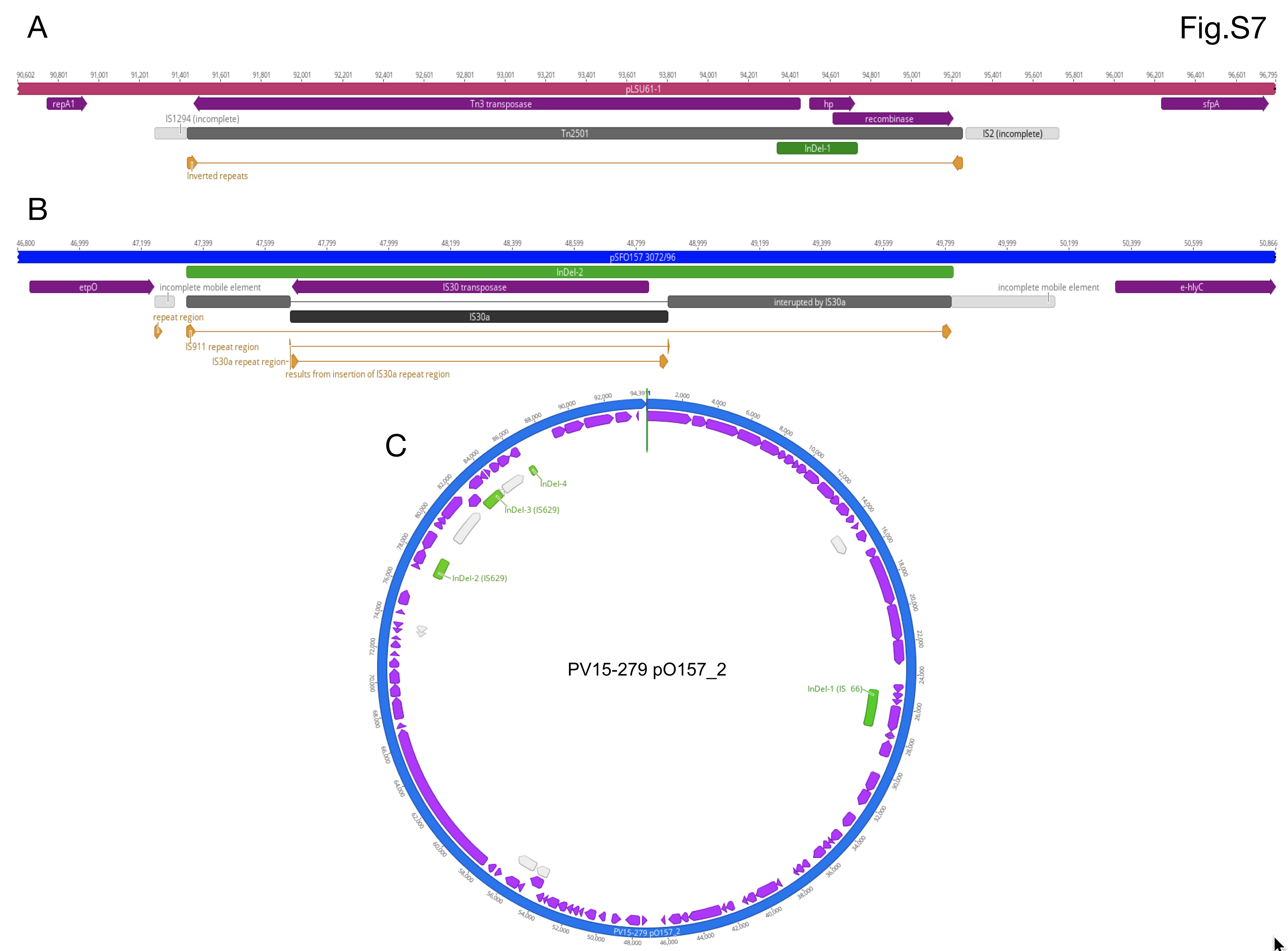

Supplement: FIGURE S7 — Length polymorphisms in plasmid pSFO157 of LSU-61 and 3072/96. Plasmid architecture and gene inventories were compared with Mauve (Darling et al., 2010), and respective annotations mapped in Geneious (Maddison and Maddison, 2016). IS elements are a major driver and hotspots for pSFO157 and pO157_2 plasmid diversification. The pSFO157 plasmid of strains LSU-61 and 3072/96 differs by 2,078 bp, (A) InDel-1 is located within the boundaries of a Tn2501 element resembling the 400 bp larger ancestral variant in LSU-61. (B) To the contrary, LSU-61 lacks a nested composite IS30/IS911 element of 2,080 bp. (C) InDels in pO157_2 of strains G5101and PV15-279, all of which are associated with insertion sequences. [file Image_7.TIFF]

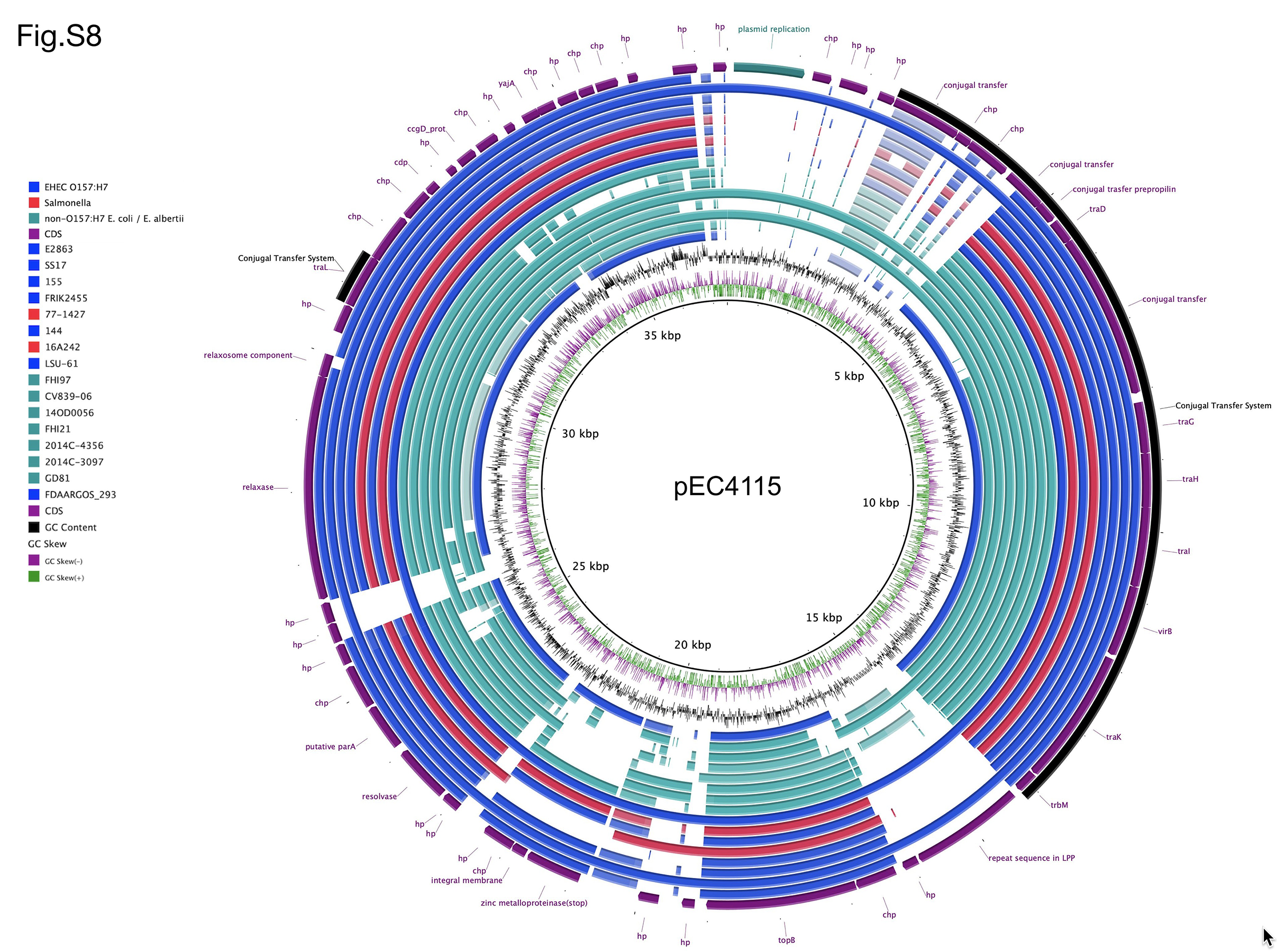

Supplement: FIGURE S8 — Comparison of pEC4115 with phylogenetically related plasmids. BRIG analysis of the plasmid architecture and gene inventory of pEC4115 and related Escherichia and Salmonella plasmids, as determined by BLASTn inferred nucleotide sequence similarities (Altschul et al., 1990). Respective gene inventories are referenced to the 37,452 bp pEC4115 plasmid in the outermost circle. CDS are shown as purple arrows and the functional annotation of loci of importance are depicted in the legend. The order of plasmids on each ring reflects the sequence similarity of query plasmids as reported by BLASTn and circle colors identify plasmid source as shown in the legend. Depicted plasmids show a considerable range in plasmid size from 34,276 bp (E. coli O103 FHI21) to 65,419 bp (Salmonella enterica). Only plasmids from SS17, and human fecal isolates O181:H49 2014C-3097 and O103 FHI21 show homology to the pEC4115 origin of replication. Noteworthy, the pEC4115 plasmid also codes conjugal transfer genes. GC-skew and GC-content of the pEC4115 reference plasmid are depicted in the two innermost circles, respectively. [file Image_8.TIFF]
